# Comparative study in estrogen-depleted mice identifies skeletal and osteocyte transcriptomic responses to abaloparatide and teriparatide

**DOI:** 10.1172/jci.insight.161932

**Published:** 2023-10-23

**Authors:** Zhengtao Lv, Jiaming Zhang, Shuang Liang, Chenhe Zhou, Dorothy Hu, Daniel J. Brooks, Mary L. Bouxsein, Beate Lanske, Paul Kostenuik, Francesca Gori, Roland Baron

**Affiliations:** 1 Division of Bone and Mineral Research, Department of Oral Medicine, Infection and Immunity, Harvard School of Dental Medicine, Boston, Massachusetts, USA.; 2 Center for Advanced Orthopedic Studies, Department of Orthopedic Surgery, Beth Israel Deaconess Medical Center, Boston, Massachusetts, USA.; 3 Harvard Medical School and Massachusetts General Hospital (MGH) Endocrine Unit, Boston, Massachusetts, USA.; 4 Radius Health, Boston, Massachusetts, USA.

**Keywords:** Bone Biology, Endocrinology, Expression profiling, Pharmacology

## Abstract

Osteocytes express parathyroid hormone (PTH)/PTH-related protein (PTHrP) receptors and respond to the PTHrP analog abaloparatide (ABL) and to the PTH 1-34 fragment teriparatide (TPTD), which are used to treat osteoporosis. Several studies indicate overlapping but distinct skeletal responses to ABL or TPTD, but their effects on cortical bone may differ. Little is known about their differential effects on osteocytes. We compared cortical osteocyte and skeletal responses to ABL and TPTD in sham-operated and ovariectomized mice. Administered 7 weeks after ovariectomy for 4 weeks at a dose of 40 μg/kg/d, TPTD and ABL had similar effects on trabecular bone, but ABL showed stronger effects in cortical bone. In cortical osteocytes, both treatments decreased lacunar area, reflecting altered peri-lacunar remodeling favoring matrix accumulation. Osteocyte RNA-Seq revealed that several genes and pathways were altered by ovariectomy and affected similarly by TPTD and ABL. Notwithstanding, several signaling pathways were uniquely regulated by ABL. Thus, in mice, TPTD and ABL induced a positive osteocyte peri-lacunar remodeling balance, but ABL induced stronger cortical responses and affected the osteocyte transcriptome differently. We concluded that ABL affected the cortical osteocyte transcriptome in a manner subtly different from TPTD, resulting in more beneficial remodeling/modeling changes and homeostasis of the cortex.

## Introduction

Parathyroid hormone (PTH) is a major endocrine regulator of skeletal homeostasis. Daily injections of its biologically active 1-34 amino acid fragment (PTH 1-34, teriparatide; TPTD) comprise the first approved anabolic therapy for the treatment of postmenopausal osteoporosis in patients with high fracture risk (1). However, despite its net bone anabolic effects driven by increased bone formation, TPTD can also stimulate bone resorption, limiting gains in bone mineral density (BMD) (2). In addition, the propensity of TPTD to increase endocortical bone remodeling can lead to increased cortical porosity, which is thought to contribute to early reductions in BMD at cortical sites (3–7). To improve the balance of bone formation and resorption and to better protect or augment cortical sites, a synthetic analog of human PTH-related protein (PTHrP) 1-34, abaloparatide (ABL), was developed. ABL clinical trials indicate its capacity to stimulate new bone formation with reduced effects on bone resorption and calcium-mobilizing potential relative to TPTD, leading to relatively greater BMD gains at cortical sites (4–8). Animal studies using different models also suggested that ABL can stimulate bone formation in a manner comparable to TPTD but with more limited induction of bone resorption and hypercalcemic effects and with better cortical bone responses (9–16). Recent in vitro studies suggest these differential actions could be due to a more transient intracellular signaling response to ABL versus TPTD (17). Since ABL and TPTD bind to the same G protein–coupled PTH receptor (PTH1R), the more transient response to ABL is thought to relate to greater relative affinity for the receptor’s “RG” conformation compared with TPTD, which is biased toward binding the “R0” conformation of PTH1R (17, 18). Because of its RG bias, ABL may induce more transient cyclic AMP responses compared with TPTD in PTH1R-expressing cells and calcemic responses in animals (17). These findings suggested that the transient cellular signaling via RG-selective binding of ABL, or differences in the endosomal recycling of the PTH1R bound to ABL or TPTD (17, 18), may favor a stronger relative anabolic effect than TPTD. Whether this is reflected in or mediated by differential regulation of gene expression in osteocytes is not known.

A range of molecular and cellular mechanisms have been proposed to explain how intermittent TPTD treatment stimulates new bone formation and decreases fracture risks in patients, with most attention focused on osteoblast responses (19–22). But PTH1R is also highly expressed in osteocytes, the most abundant cell in bone, and its expression in these cells is required for normal skeletal response to intermittent or continuous TPTD treatment (23–26). Osteocytes are also key orchestrators of bone remodeling (27–30), suggesting that their responses to bone-targeted treatments may be central to the overall skeletal response. Indeed, although osteocytes are embedded in bone matrix, these terminal descendants of osteoblasts express several membrane-localized or secreted paracrine factors, including PTHrP, that regulate osteoblast and osteoclast activity (31, 32). Osteocytes also secrete hormones that affect mineral metabolism within and beyond the skeleton, including Fgf23 (31). Other osteocyte-secreted factors that play key roles in regulating bone remodeling and skeletal homeostasis include TNF superfamily member 11 (Rankl) and TNF receptor superfamily member 11b (Opg), the main regulators of osteoclast differentiation and bone resorption, and sclerostin (encoded by the *Sost* gene), which inhibits bone formation and favors bone resorption by reducing canonical Wnt signaling (33). PTH1R-mediated stimulation of bone formation and bone resorption appears to arise in part from a local decrease in sclerostin and increase in Rankl (25). Although ABL and TPTD were reported to similarly regulate *Sost* and *Rankl* (34), whether ABL and TPTD differentially affect the osteocyte transcriptome and whether this could explain the differential responses in cortical bone are not known. We therefore focused our mechanistic investigations on determining whether the relative effects of ABL versus TPTD on cortical bone relate to differential osteocyte responses.

To accomplish this goal, we compared the skeletal and the osteocytic responses to ABL and TPTD treatment in the ovariectomized (OVX) model in mice. Although ABL and TPTD similarly prevented trabecular bone loss after ovariectomy, ABL had greater overall effects on cortical bone versus TPTD. Interestingly, both peptides significantly decreased osteocyte lacunar area (LcA) in sham-operated and OVX animals, suggesting that ABL and TPTD induced a positive bone balance during peri-lacunar remodeling (PLR), similar to their effects on bone surface (BS) remodeling. Finally, the transcriptomes of cortical osteocyte-enriched preparations from ABL- and TPTD-treated animals showed substantial overlap but also differed in some of the genes they targeted and in their effects on common target genes and signaling pathways.

## Results

### Both TPTD and ABL treatment protect from ovariectomy-induced trabecular bone loss.

A total of 51 mice (8–10/group) underwent bilateral sham or ovariectomy surgery at 12 weeks of age. Seven weeks postsurgery (age of 19 weeks), mice were injected daily subcutaneously (SC) for 4 weeks with 0.9% normal saline (VEH), ABL, or TPTD at 40 μg/kg/d. Mice were sacrificed at 23 weeks of age, 18–24 hours after the final peptide injection (Supplemental Figure 1A; supplemental material available online with this article; https://doi.org/10.1172/jci.insight.161932DS1). Successful ovariectomy surgery was confirmed in each mouse at necropsy by reduced uterine weights versus sham controls (Supplemental Figure 1B). The OVX group also showed an expectedly greater final body weight relative to sham groups (Supplemental Figure 1B). Intriguingly, whereas the treatments had no significant effects on body weight in sham mice, TPTD or ABL mitigated increases in body weight in the OVX mice (Supplemental Figure 1B).

Two-way ANOVA showed highly significant effects of both treatments on bone microstructure by μCT and histomorphometry in vertebra trabecular bone (Supplemental Figures 2 and 3 and Supplemental Table 1). Overall, however, TPTD and ABL had similar effects on vertebra trabecular bone in both sham and OVX animals (Supplemental Figures 2 and 3 and Supplemental Table 1). In the sham animals, bone volume/total volume (BV/TV) was significantly increased as was BMD, but this latter difference was significant only with ABL (Supplemental Figure 2 and Supplemental Table 1). The number of osteoblasts (N.Ob) and number of osteoclasts (N.Oc) were both significantly increased, validating an overall increase in trabecular turnover with both treatments (Supplemental Figure 3 and Supplemental Table 1).

In the OVX animals treated with vehicle, BV/TV was expectedly lower than in the sham group, and was increased by both treatments, though again BMD was significantly increased only in the ABL group (Supplemental Figure 2 and Supplemental Table 1). Surprisingly, although bone formation rate (BFR) and N.Ob were significantly increased in both treated groups, the N.Oc, significantly increased in the OVX group, was not further modified by treatment, in contrast with the effects observed in the sham group. ABL did not show, at this time point after ovariectomy, a different N.Oc compared to TPTD (Supplemental Figure 3 and Supplemental Table 1). Thus, as expected, ABL and TPTD have similar effects on trabecular bone remodeling and microstructure in sham and in OVX animals.

### ABL has a stronger anabolic effect than TPTD on cortical bone.

**Figure 1:**
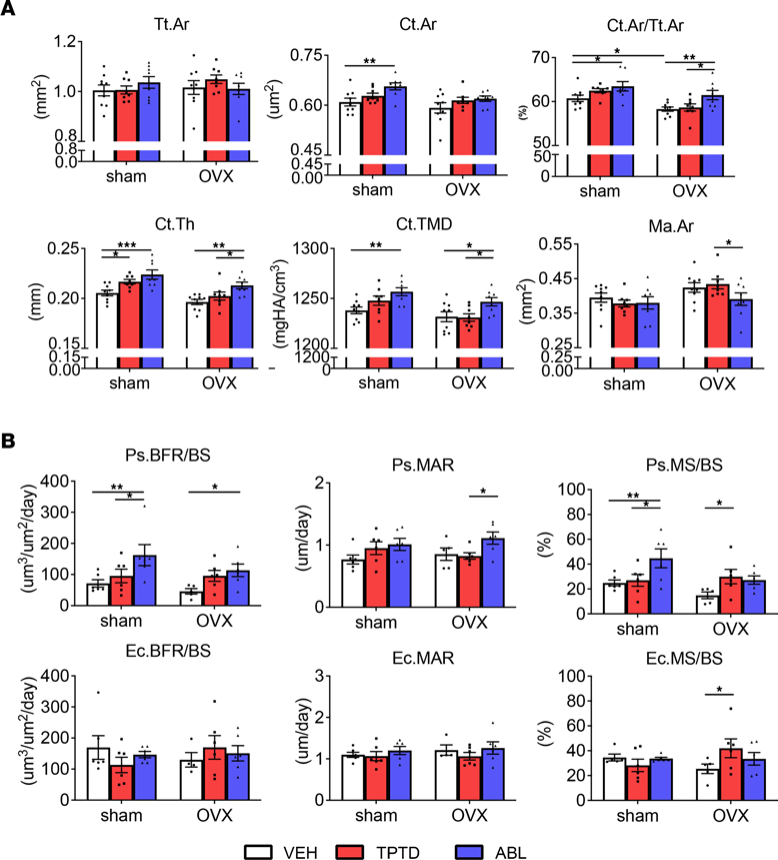
Cortical bone responses. (**A**) μCT analysis and (**B**) dynamic bone histomorphometry analysis of tibia midshaft in sham and OVX groups treated with VEH, TPTD, or ABL. Data are expressed as mean ± SEM. Individual dots represent the number of mice in each group. Two-way ANOVA followed by Fisher’s least significant difference (LSD) test. *P < 0.05, **P < 0.01, ***P < 0.001. Tt.Ar, total area; Ct.Ar, cortical area; Ct.Th, cortical thickness; Ct.TMD, cortical tissue mineral density; Ma.Ar, marrow area; Ps., periosteal; MS/BS, mineralized surface/bone surface; MAR, mineral apposition rate; Ec., endocortical.

In contrast with trabecular bone, TPTD and ABL elicited different responses in cortical bone (Figure 1). Ovariectomy affected several cortical parameters at the tibia diaphysis: μCT analysis showed that Ma.Ar was significantly greater and Ct.Th lower in the OVX-VEH group compared with sham-VEH, consistent with increased endocortical resorption (Figure 1A and Table 1). After treatment, the OVX-ABL group had significantly smaller Ma.Ar than the OVX-TPTD group, whereas both ABL and TPTD groups had higher Ct.Th compared with OVX-VEH (Figure 1A and Table 1). The OVX-ABL group had greater Ct.Ar/Tt.Ar and Ct.TMD versus the OVX-TPTD group (Figure 1A and Table 1). Dynamic cortical bone histomorphometry revealed no differences between sham-VEH and OVX-VEH for periosteal and endocortical parameters (Figure 1B and Table 1). However, Ps.BFR/BS was significantly higher in the ABL groups than both sham-VEH and OVX-VEH groups and significantly higher than TPTD in the sham group (Figure 1B and Table 1). The same was true for Ps.MS/BS. Ec.BFR/BS and Ec.MAR were similar in all groups. Ec.MS/BS was higher in the OVX-TPTD group versus OVX-VEH controls (Figure 1B and Table 1).

Thus, analysis of changes in cortical bone revealed significant differences between TPTD and ABL: Ct.Th was increased in both groups, but the Ma.Ar was significantly smaller in the ABL-treated versus TPTD-treated OVX group, such that cortical area/total area and Ct.TMD were significantly higher in the ABL-treated sham and OVX groups than VEH-treated animals and TPTD-treated animals (Figure 1A and Table 1).

### Biochemical markers of bone turnover.

Consistent with the histomorphometry results, 11 weeks post-ovariectomy led to a relatively modest increase in bone turnover markers, such that at time of sacrifice, OVX resulted only in a small increase in both C-terminal telopeptide of type I collagen (CTX) and procollagen type I N-propeptide (PINP) (Supplemental Table 2). Ovariectomy had a significant effect on the serum bone formation marker P1NP, but 4 weeks of peptide administration did not have significant effects in both sham and OVX groups on either CTX or P1NP (2-way ANOVA, Supplemental Table 2), despite measurable histomorphometric effects on the N.Oc in sham and BFR in OVX animals (Supplemental Figure 3 and Supplemental Table 1).

### Effects of TPTD and ABL on long bone biomechanics.

Destructive 3-point bending tests of femurs indicated no between-group differences in most biomechanical properties. Only apparent ultimate stress was significantly higher in the sham-TPTD and sham-ABL versus sham-VEH controls (Supplemental Figure 4A and Supplemental Table 3). No difference was detected between the 2 treatments.

Correlations between μCT-derived femoral structural parameters and ultimate moment were evaluated using linear regression analysis (Supplemental Figure 4B). Femoral diaphyseal ultimate moment was strongly correlated with femoral Ct.Ar across all groups combined (*r* = 0.79, *P* < 0.0001), as well as within the TPTD group (*r* = 0.91, *P* < 0.0001) and the ABL group (*r* = 0.69, *P* = 0.003). Femoral diaphyseal Ct.Th was also a strong predictor of ultimate moment among all groups (*r* = 0.64, *P* < 0.0001), the TPTD group (*r* = 0.72, *P* = 0.0017), and the ABL group (*r* = 0.59, *P* = 0.0155). Femoral diaphyseal ultimate load also correlated with femoral Ct.TMD, with an overall *r* value of 0.60 (*P* = 0.0003) across all groups and an *r* value of 0.68 (*P* = 0.00041) in the ABL group and *r* = 0.53 (*P* = 0.0332) for the TPTD group. Overall, this indicates that material properties were not altered by the treatments, but no significant difference was observed between TPTD and ABL treatment.

### TPTD and ABL treatment alter osteocyte PLR in favor of matrix accrual.

Our findings support the concept that, even if used at the same dosage as TPTD, ABL has an overall more favorable effect than TPTD on cortical bone, on both the periosteal and endosteal surfaces. Given that the largely predominant cell type in cortical bone is the osteocyte, we then explored structural and transcriptomic changes in that cell population.

**Figure 2:**
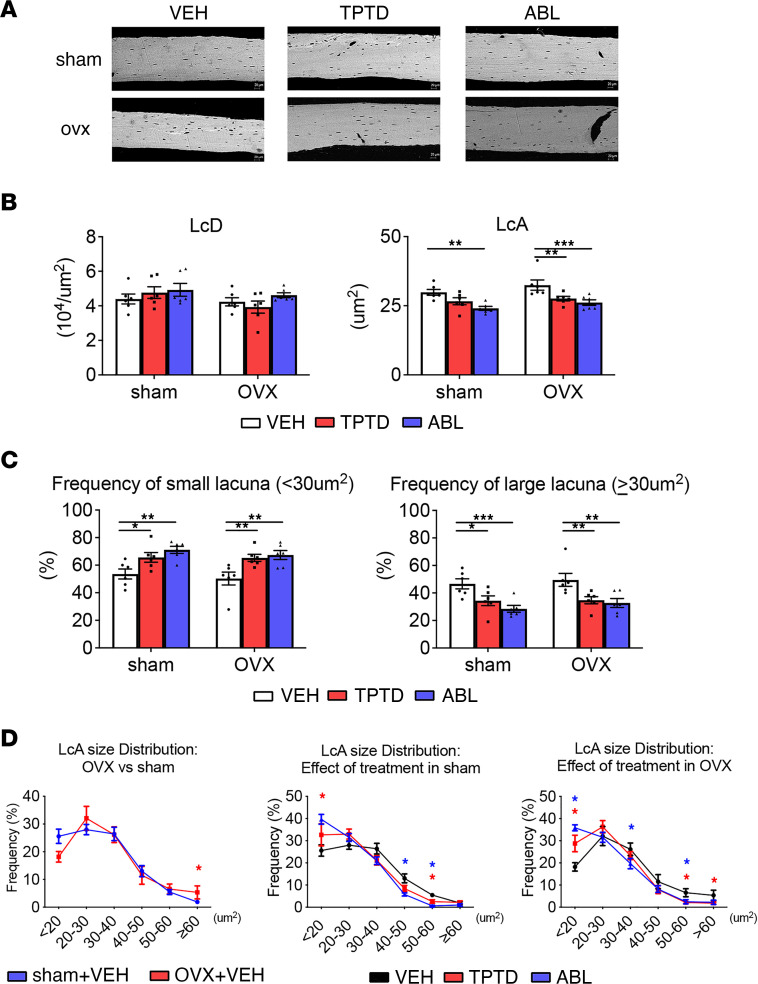
BSEM analysis of osteocyte lacunae. (**A**) Representative BSEM images (original magnification, ×500) of the femur diaphysis. Scale bars: 20 μm. (**B**) Measurements of single osteocyte lacunar density (LcD) and lacunar area (LcA). (**C**) Frequency of small (<30 μm^2^) and large lacunae (≥30 μm^2^) of osteocyte LcA in sham and OVX groups treated with VEH, TPTD, or ABL. Individual dots represent the number of mice in each group. (**D**) Size distribution of osteocyte lacunar area in sham and OVX groups treated with VEH, TPTD, or ABL. Individual dots represent the number of mice in each group. Two-way ANOVA followed by Fisher’s LSD test. *P < 0.05, **P < 0.01, ***P < 0.001. Red* and blue* indicate significance of the TPTD- and ABL-treated groups, respectively, compared with VEH groups. BSEM, backscatter scanning electron microscopy.

Overall, as expected for a relatively short experimental period relative to cortical remodeling, the density of osteocyte lacunae (LcD) of the tibia cortical midshaft region of interest remained unchanged after ovariectomy and was not significantly altered by treatments (Figure 2A and Supplemental Table 4). The mean osteocyte lacunar area (LcA) was 8.7% larger in the OVX group than in the sham group (Figure 2B). Although this overall difference did not reach significance, analysis of the size distribution of the lacunae demonstrated a significant increase in the number of large osteocyte lacunae (≥60 mm^2^) and a decrease in small osteocyte lacunae <20 mm^2^ (Figure 2, C and D, and Supplemental Table 4), clearly suggesting an ovariectomy-dependent change in PLR leading to an enlargement of osteocyte lacunae.

Strikingly, both sham and OVX mice treated with ABL or TPTD showed significantly smaller LcA compared with their respective sham-VEH and OVX-VEH controls. No significant differences were found between the ABL and the TPTD treatment in both the sham and OVX groups. Two-way ANOVA of the frequency of osteocyte LcA showed an overall significantly increased frequency of lacunae with the smallest area (<20 mm^2^) in the OVX-ABL and OVX-TPTD groups compared with the OVX-VEH group (Supplemental Table 4 and Figure 2, C and D), together with a decrease in the frequency of osteocyte lacunae with an area ≥ 30 mm^2^ (Figure 2, C and D, and Supplemental Table 4). Thus, chronic treatment with TPTD or ABL altered osteocytic PLR in favor of matrix accrual, and no significant quantitative differences were observed between the 2 treatments. We then determined whether ABL and TPTD elicited different signaling events in an osteocyte cell line (Ocy454) and/or changes in the transcriptome of cortical osteocytes.

### Comparison of the osteocyte signaling and gene expression responses to TPTD and ABL.

Given the changes observed in the osteocyte lacunae upon treatment, we examined the osteocyte responses at the molecular level by 2 approaches. First, Ocy454 cells were treated with TPTD or ABL in vitro and assessed for proximal signaling changes; second, bulk RNA-Seq was performed on enriched primary cortical osteocyte preparations isolated from long bones of sham and OVX mice treated with vehicle, TPTD, or ABL.

**Figure 3:**
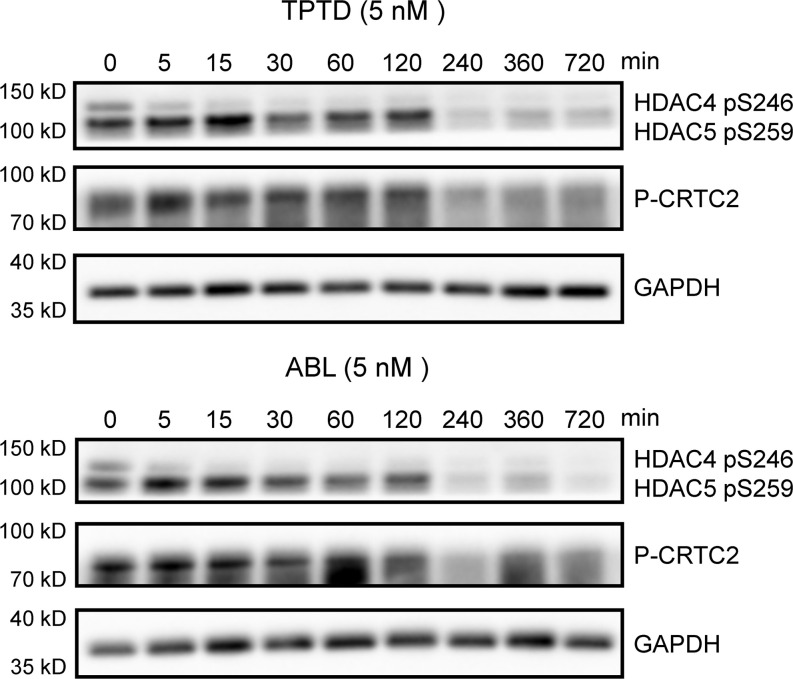
PTH1R signaling. Representative Western blots for HDAC4/5 and CRCT2 phosphorylation in Ocy454 cell line treated with or without TPTD (5 nM) or ABL (5 nM) for the time indicated. GAPDH was used as loading control (*n* = 3).

Early signaling events after activation of the PTH1R were monitored in vitro for up to 3 hours after treatment of Ocy454 cells with 5 nM TPTD or ABL. In particular we assessed changes in the phosphorylation levels of histone deacetylase 4/5 (Hdac4/5) and CREB regulated transcription coactivator 2 (Ctrc2), previously shown to regulate *Sost* and *Rankl* expression in these cells upon PTH signaling activation (34). Confirming previous findings (34), we did not detect any differences between ABL and TPTD, used at the same concentration, in the phosphorylation levels and in the dynamics of phosphorylation of Hdac4/5 or Ctrc2 (Figure 3), suggesting that events proximal to the PTH1R receptor are essentially similar in amplitude and time course after ABL or TPTD binding to the PTH1R, at least for the pathways we have examined.

To determine whether signaling and transcriptional events involving other targets distal to receptor activation differed between these 2 molecules, we then analyzed osteocyte gene expression profiles. Osteocyte enrichment in our samples was monitored through the relative expression of the osteocyte marker gene *Sost* (32, 35, 36) and *Kera* (keratocan), which encodes for a proteoglycan highly expressed by osteoblasts but not by osteocytes (37). *Sost* expression was enriched 15-fold relative to the osteoblast-enriched population in the same samples, whereas the expression of *Kera* was significantly depleted in the osteocyte-enriched population compared with the osteoblast-enriched population, verifying successful enrichment for osteocyte-derived RNA in these samples (Supplemental Figure 5).

### Ovariectomy alters gene expression in cortical osteocytes.

**Figure 4:**
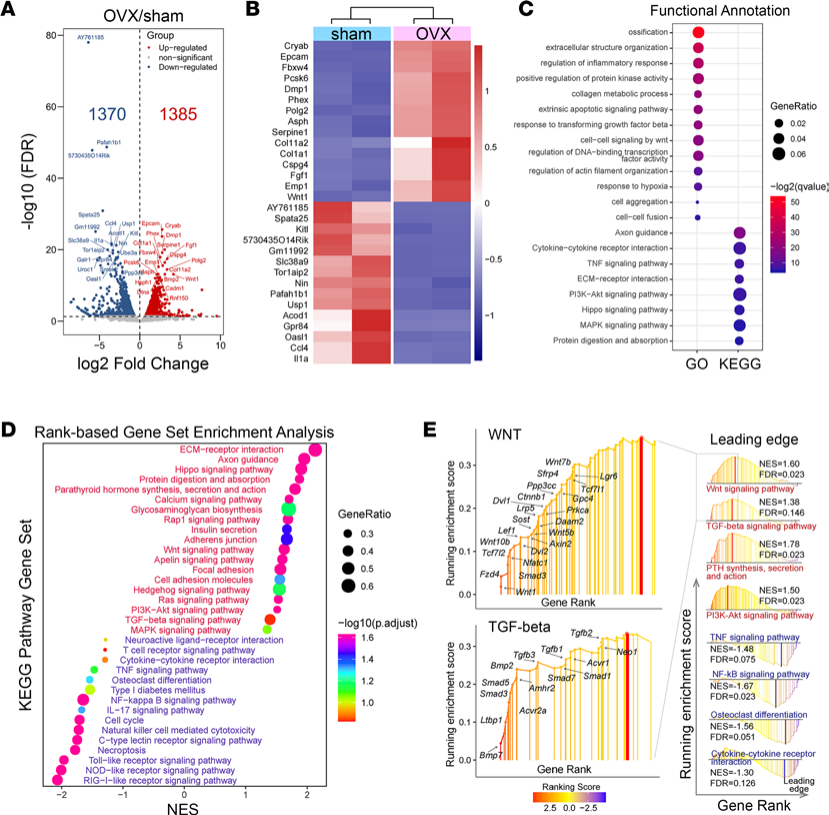
Bulk RNA-Seq. Effect of ovariectomy on the osteocyte gene landscape and signaling pathways. (**A**) DEGs in OVX versus sham (red and blue dots represent up- or downregulated genes, FDR < 0.05) osteocyte-enriched samples. (**B**) Heatmap of top 15 differentially regulated genes according to FDR. (**C**) Functional annotation of the DEGs by GO and KEGG enrichment analyses. GeneRatio indicates the gene number ration in each GO function and KEGG pathway. The color and size of dots represent *P* value adjusted by Benjamini-Hochberg method and gene number assigned to the corresponding GO term and KEGG pathway, respectively. (**D**) Gene set enrichment analysis. (**E**) Leading-edge analysis of pathways critical for bone biology. DEGs, differentially expressed genes; GO, Gene Ontology; KEGG, Kyoto Encyclopedia of Genes and Genomes; NES, normalized enrichment score.

Using these highly enriched osteocyte RNA preparations, we first looked at the effects of ovariectomy on the osteocyte gene expression profile (Figure 4 and Supplemental Figure 6). As expected, the osteocyte gene expression landscape was markedly altered by ovariectomy. We identified 2,755 differentially expressed genes (FDR < 0.05), of which 1,385 genes were upregulated and 1,370 downregulated upon ovariectomy (Figure 4A). Among these 2 groups, 984 upregulated genes and 1,045 downregulated genes had a log_2_FC > 1 or < –1 (74% of all differentially regulated genes). As shown in the heatmap (Figure 4B), among the 15 top regulated genes, we identified Dmp1 and Phex, highly expressed by osteocytes (27, 38), the expression of which was significantly higher after ovariectomy. GO and KEGG analysis of genes with FDR < 0.05 and log_2_FC > 1 or < –1, identified genes associated with ossification, extracellular matrix, collagen metabolic process, and cytokine-cytokine receptor interaction (Figure 4C). PTH signaling, calcium signaling, PI3K/AKT signaling, TGF-β signaling, and Wnt signaling were among the top ranked KEGG signaling pathways upregulated in response to ovariectomy (Figure 4, D and E), while TNF, NF-κB, and cytokine-cytokine receptor signaling were among the top ranked KEGG signaling pathways downregulated by ovariectomy in osteocyte-enriched samples (Figure 4, D and E). Among the genes belonging to the Wnt signaling pathway, *Wnt1* was the most upregulated gene whereas several other genes known to be involved in Wnt signaling and to regulate skeletal homeostasis (*Lrp5*, *Lrp6*, *Ctnnb1*, *Wnt5b*, *Wnt7b*, *Wnt10b*, *Tcf1*, *Axin*, *Lef1*, *Dvl2*, *Fzd4*, *Sost*, and *Sfrp4*) (39) were upregulated by ovariectomy (Figure 4E), suggesting an overall activation of Wnt signaling, both canonical and noncanonical, after ovariectomy. Similarly, several genes of the TGF-β superfamily (*Bmp*; *Bmp7*; *Smad1*, -*3*, -*5*, and -*7*; and *Tgfb1–3*) were increased by ovariectomy (Figure 4E). Axon guidance, a pathway involved in osteocyte network formation (40–42), was also upregulated and was among the top ranked KEGG pathways affected by ovariectomy (Figure 4, C and D). Surprisingly, but consistent with the histomorphometry findings, gene set enrichment analysis (GSEA) showed that the osteoclast differentiation pathway was significantly downregulated in osteocytes at the delayed time point used here after ovariectomy, although *Nfatc1* was increased (Figure 4, D and E, and Supplemental Figure 6). These results show that osteocytes respond to ovariectomy with changes that may affect the regulation of bone and PLR in this OVX model.

### TPTD and ABL alter osteoclast- and osteocyte-associated gene sets in cortical osteocyte-enriched preparations.

**Figure 5:**
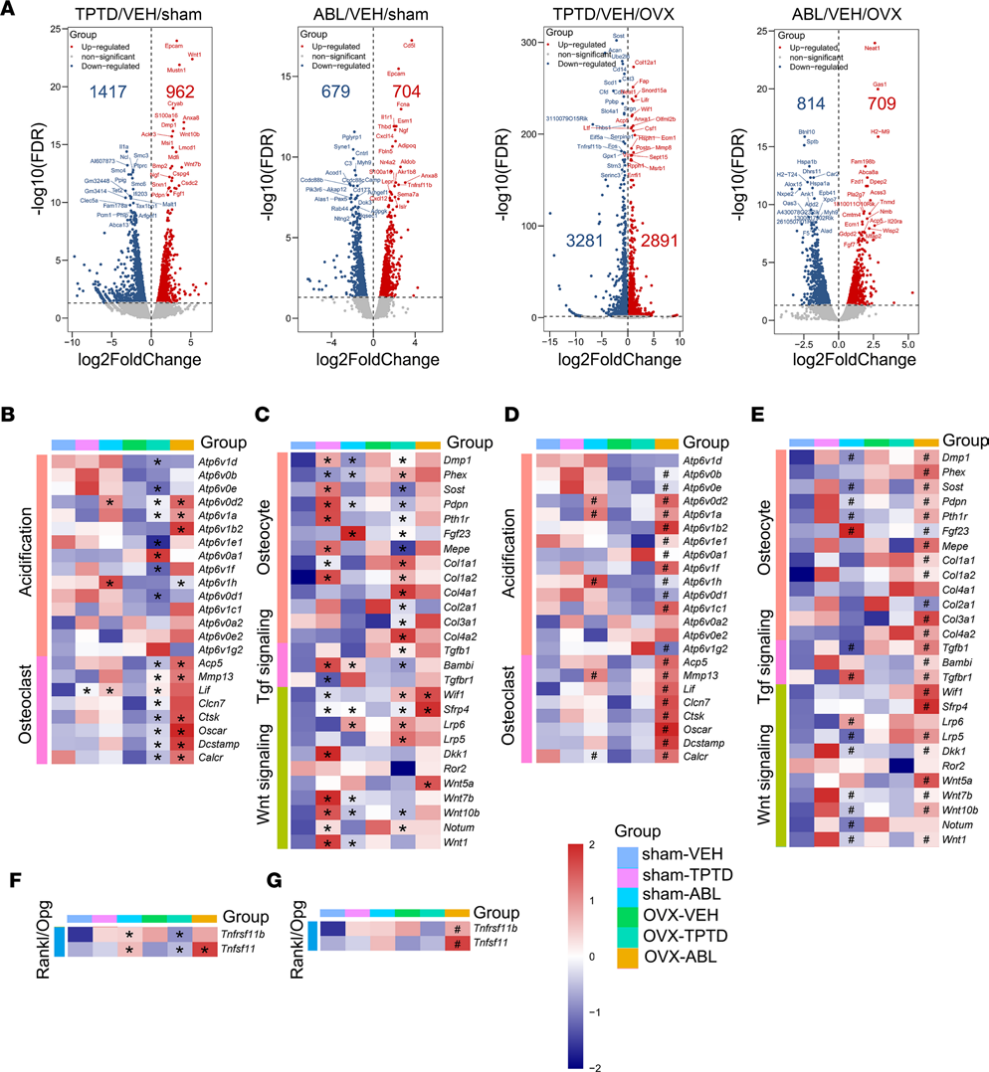
Bulk RNA-Seq. TPTD- or ABL-regulated genes in sham and OVX groups. (**A**) Volcano plots of DEGs (FDR < 0.05) in TPTD or ABL treatment compared with VEH. The top 20 upregulated or downregulated genes induced by TPTD or ABL were labeled according to FDR. (**B**–**G**) Comparisons between treatments (TPTD or ABL) and VEH in sham or OVX groups. (**B**–**F**) *FDR estimated by DESeq2 or EdgeR and between ABL and TPTD in sham or OVX groups; (**D**–**G**) ^#^FDR estimated by DESeq2 or EdgeR.

We then probed RNA-Seq databases to establish the specific signatures of TPTD- and of ABL-regulated genes in sham and OVX mice. As shown by volcano plots (Figure 5A), in sham animals, TPTD treatment differentially regulated 2,379 (FDR < 0.05) genes, of which 962 were upregulated and 1,417 downregulated. ABL treatment resulted in 1,383 (FDR < 0.05) differentially regulated genes, of which 704 were upregulated and 679 downregulated (Figure 5A). In OVX mice, we identified 6,172 (FDR < 0.05) differentially regulated genes by TPTD, of which 2,891 were upregulated and 3,281 downregulated (Figure 5A), whereas 1,523 genes (FDR < 0.05) were differentially regulated by ABL with 709 genes upregulated and 814 downregulated (Figure 5A).

Both TPTD and ABL treatment, and in both sham and OVX mice, led to a significant enrichment in the expression of genes belonging to the Wnt and TGF-β signaling pathways, over and above the activation already observed after ovariectomy (Supplemental Figure 7). We then examined specific gene sets corresponding to osteocyte and bone formation markers, bone resorption markers, and osteoclast-regulating cytokines and signaling pathways (Figure 5, B–G). Since treatments appeared to differentially influence osteoclast numbers in the sham versus OVX groups (higher in the former) at the chosen time point (Table 1), we analyzed osteocyte-expressed osteoclast-regulating cytokines in the sham and OVX groups separately. As shown in Figure 5, F and G, in the sham group both treatments increased *Tnfsf11* (*Rankl*) and *Tnfrsf11b* (*Opg*) levels compared with vehicle-treated group. In the OVX group, TPTD and ABL increased *Tnfsf11* levels, while only TPTD decreased *Tnfrsf11b* levels compared with the OVX-VEH–treated group. Importantly, ABL treatment significantly increased both *Tnfsf11* and *Tnfrsf11b* levels compared with TPTD treatment.

The same was true for a complete set of resorption marker genes involved in acidification (*Atp6v* subunits) or other osteoclast markers (*Dcstamp*, *Acp5*, *Ctsk*, *MMP13*, *Lif*, *Clcn7*, *Oscar*, *Calcr*) (Figure 5, B and D). Importantly, the expression of this entire gene set was significantly different, and often substantially higher, in the ABL group than in the TPTD group, particularly after ovariectomy (Figure 5, B and D). Given that we have been studying highly enriched osteocyte RNA (Supplemental Figure 5), this suggests that, in response to TPTD or ABL treatment, osteocytes alter their phenotype by increasing their expression markers and regulators of resorption.

We then examined a set of genes and signaling pathways reflecting the osteocyte phenotype and bone matrix formation (Figure 5, C and E). Overall (Figure 5C), treatment with TPTD or ABL altered significantly the expression profile of this gene set, but as shown in Figure 5E, ABL altered the expression of these genes in a significantly different manner from TPTD in both sham and OVX groups. We found similar trends in the regulation of key Wnt genes. As previously reported in vitro (12), *Dkk1* was significantly upregulated by TPTD but not by ABL in the sham group, although this difference was not apparent in the OVX group (Figure 5C). Importantly, several genes reflecting bone matrix collagen production were increased significantly by both treatments, but particularly with TPTD (Figure 5C), with ABL significantly different from TPTD, especially in the OVX group (Figure 5, C and E). Surprisingly, ABL induced more robust increases in osteoclast-related genes (Figure 5, B and D), cytokines regulating osteoclast differentiation (Figure 5, F and G), and osteoblast/osteocyte-related genes (Figure 5, C and E).

Thus, this analysis indicates that if they altered the expression of bone resorption and bone formation genes in osteocytes in similar ways, the effect of ABL was often significantly different from that of TPTD, in amplitude and/or in direction relative to vehicle-treated groups. We then proceeded to identify differentially expressed genes between TPTD and ABL.

### Comparative effect of ABL and TPTD on other genes in the osteocyte transcriptome: identification of genes responding uniquely to ABL.

**Figure 6:**
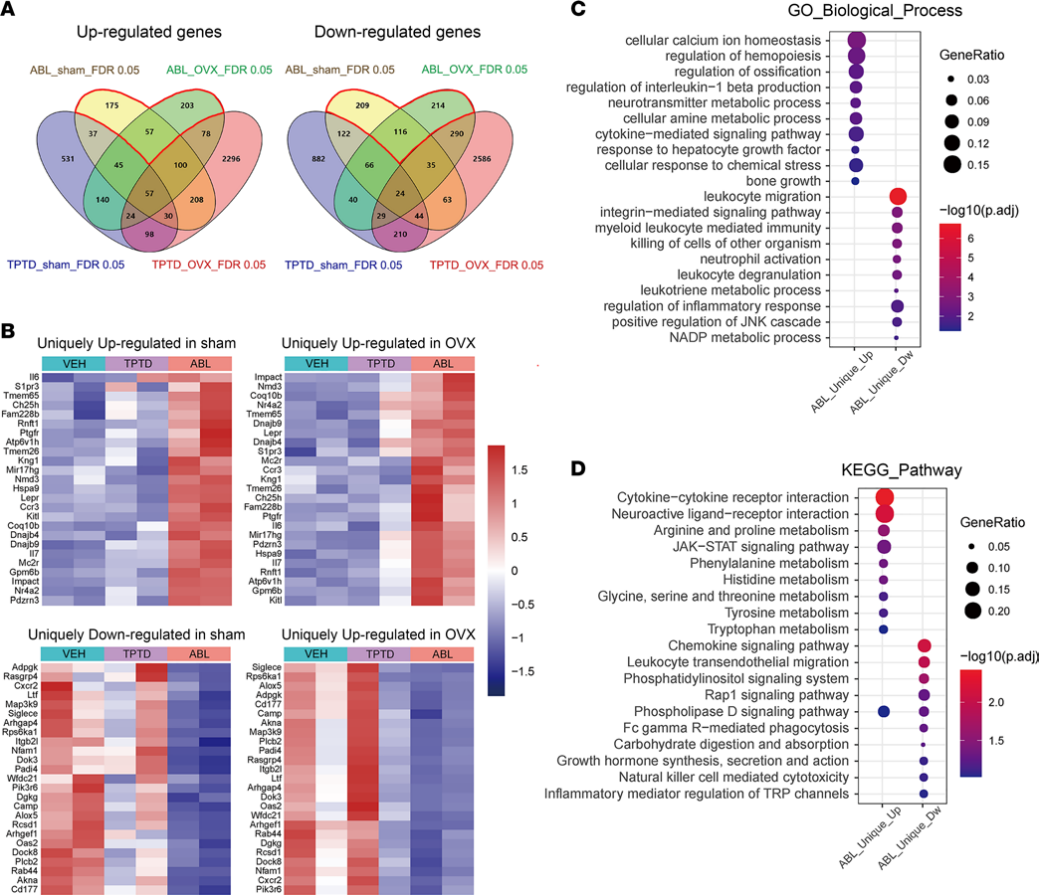
Bulk RNA-Seq. Specific signatures of ABL-regulated genes in sham and OVX groups. (**A**) Venn diagrams of 4 sets of genes regulated by TPTD or ABL in sham or OVX groups. (**B**) Heatmaps of the unique top 25 genes up- or downregulated by ABL. (**C** and **D**) Functional annotation of ABL-unique regulated genes by GO (**C**) and KEGG (**D**) enrichment analyses. GeneRatio indicates the gene number ratio in each GO function and KEGG pathway. The color and size of dot represent *P* value adjusted by Benjamini-Hochberg method and gene number assigned to the corresponding GO term and KEGG pathway, respectively.

The 2 Venn 4–gene set diagrams shown in Figure 6A illustrate the simple set of relations and overlaps between the genes uniquely or commonly regulated by the 2 treatments in sham and OVX animals. To determine whether, relative to TPTD, ABL affects a specific set of genes within the genome-wide osteocyte transcriptome, we extracted from the RNA-Seq database the genes and signaling molecules uniquely regulated by ABL.

**Figure 7:**
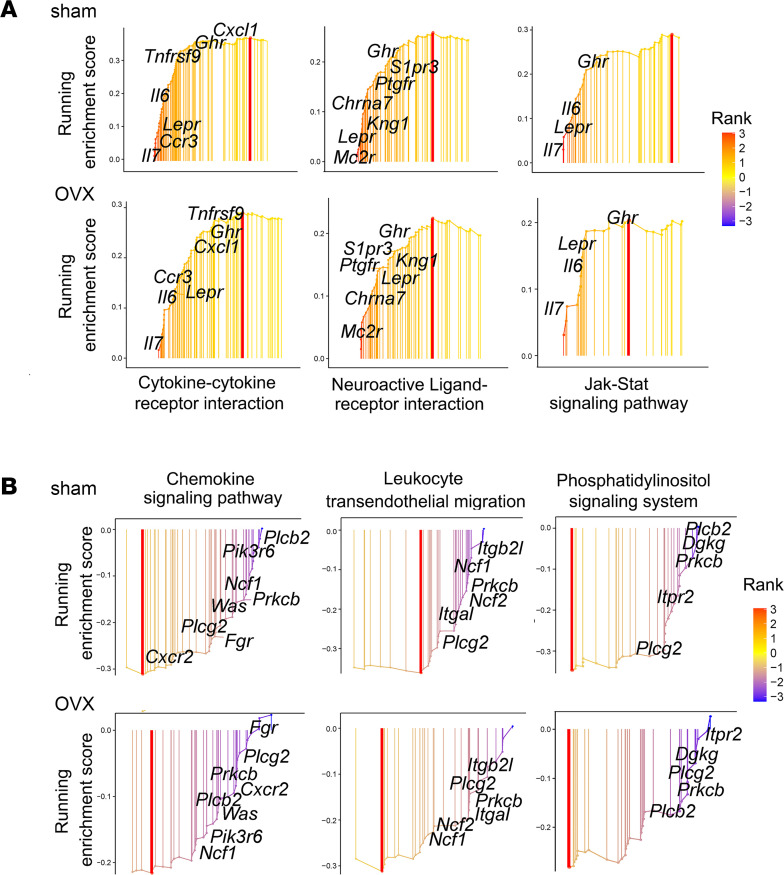
Bulk RNA-Seq. Signaling pathways and genes uniquely regulated by ABL. (**A**) Upregulated signaling pathways include the cytokine-cytokine receptor interaction, neuroactive ligand-receptor interaction, and JAK/STAT pathway. *Lepr* and *Ghr* are 2 common genes involved in these 3 pathways. (**B**) Downregulated signaling pathways include the chemokine signaling pathway, leukocyte transendothelial migration, and phosphatidylinositol signaling system. *Prckb* and *Plcg2* are 2 common genes involved in these 3 pathways.

As shown in Figure 6A, a total of 435 genes were uniquely upregulated by ABL in sham mice, of which 175 were observed only in the sham, 203 only in the OVX mice, and 57 genes in both groups. Of the downregulated genes, 539 were uniquely regulated by ABL, of which 209 were observed only in the sham, 214 only in the OVX mice, and 116 genes in both groups. Given that our study is focused on bone responses and that the effects of treatment on bone remodeling and osteocyte lacunae were generally similar in sham and in OVX animals (Figures 1 and 2 and Table 1), with the exception of the response of osteoclasts (Supplemental Figure 3 and Supplemental Table 1), we then focused on the set of ABL-unique genes that were similarly upregulated (numbering 57) or downregulated (numbering 116) in both sham and OVX group osteocytes (Figure 6, A and B). Among these “ABL-specific” genes, GO analysis showed an enrichment in genes associated with biological processes such as cellular calcium ion homeostasis, regulation of ossification, and bone growth (Figure 6C). Noticeably, KEGG pathway analysis revealed that the 3 pathways most uniquely upregulated by ABL were the cytokine-cytokine receptor interaction, the neuroactive ligand-receptor interaction, and the JAK/STAT signaling pathway and this in both the sham and the OVX context (Figure 6D and Figure 7A). The 3 pathways most uniquely downregulated by ABL were the chemokine signaling pathway, the leukocyte transendothelial migration, and the phosphatidyl/inositol signaling system, again independent of the sham or OVX context (Figure 6D and Figure 7B). All 3 of the downregulated pathways included the gene encoding PLCg2, an important signaling enzyme that catalyzes the conversion of 1-phosphatidyl-1D-myo-inositol 4,5-bisphosphate (IP2) to 1D-myo-inositol 1,4,5-trisphosphate (IP3) and diacylglycerol using calcium as a cofactor. Extraction of the leading-edge genes from these ABL-regulated pathways identified genes with an established role in skeletal biology, bone remodeling, and postmenopausal osteoporosis and in immune-mediated bone diseases, including rheumatoid arthritis, as well as in the crosstalk between cells involved in bone homeostasis and hematopoiesis, such as the leptin receptor (*Lepr*), interleukin-6 (*Il6*), interleukin-7 (*Il7*), sphingosine 1 phosphate receptor 3 (*S1pr3*), growth hormone receptor (*Ghr*), melanocortin 2 receptor (*Mc2r*), CC chemokine receptor 3 (*Ccr3*), CXC chemokine 1 (*CxCl1*), and CXC chemokine receptor 2 (*Cxcr2*) (Figure 7). Furthermore, several genes with no known relationship to bone biology were also identified as up- or downregulated specifically by ABL and will constitute an interesting starting point for further studies on the specific responses to ABL.

## Discussion

ABL is a synthetic 34–amino acid peptide analog of human PTHrP 1-34, approved for the treatment of postmenopausal osteoporosis patients at high risk of fracture. It is the second PTH-like agent to be approved for osteoporosis management, after teriparatide. Studies in women and men with osteoporosis indicate that ABL can stimulate bone formation in a manner comparable to TPTD but with less induction of bone resorption and reduced hypercalcemic effects. Of particular interest to this report are the differences between ABL and TPTD in their effects on cortical bone, where TPTD increases markedly endosteal bone remodeling, initially decreasing BMD and increasing porosity (3–7, 43), whereas ABL has a more favorable effect on modeling bone formation at the cortical endosteum and periosteum, in both human and animal studies (14, 44). The aim of this study was to verify these differences and attempt to get some insight into their molecular and cellular basis. We therefore examined the responses of cortical osteocytes, which express the PTHR1, respond to PTH treatment in vivo, and are important regulators of bone remodeling and homeostasis (28, 30–32, 45, 46).

Our results show that in mice, even though treatment was initiated late after ovariectomy (7 weeks), ABL and TPTD led to substantial improvements in vertebral trabecular bone volume and bone formation parameters. The effects of the 2 treatments were very similar. Surprisingly, in the OVX group neither ABL or TPTD showed increased osteoclast numbers, nor did they show increases in the serum bone resorption marker CTX. We interpret these data to show that 7 weeks after ovariectomy, a time at which bone volume was low, turnover has already markedly decreased, as previously observed (58, 61, 57). We cannot exclude the possibility that differences in resorption occurred at earlier time points, as suggested by the changes observed in midshaft marrow area (see below). In contrast, the effects of treatment on osteoclasts are significant in the sham groups, where the expected increase in the number of osteoclasts was observed. Again, there were no significant differences between ABL and TPTD.

In contrast, we found the effects on cortical bone to be significantly different, with ABL providing better protection than TPTD after ovariectomy. Our findings show that even at the same dose, ABL showed significantly better responses on several parameters in the cortex, particularly in OVX mice. Both treatments significantly increased cortical thickness, but ABL had a significantly greater effect on several cortical parameters, increasing cortical area, cortical BMD, and periosteal apposition while significantly decreasing marrow area (Figure 1 and Table 1). In cortical bone the first response to estrogen withdrawal is enhanced endosteal resorption, which leads to increased marrow area and decreased bone mass and strength (47). Notably here, only ABL was able to prevent the increase in marrow area, showing even significantly smaller marrow area than the OVX-VEH–treated group (Figure 1 and Table 1). Thus, it appears that ABL prevented the ovariectomy-induced endocortical expansion more effectively than TPTD in this model, consistent with several reports indicating less osteoclast induction with ABL than with TPTD. Furthermore, although all groups showed an increase in periosteal bone formation, only ABL showed significant increases in MAR, MS, and BFR on this bone surface (Figure 1 and Table 1), confirming previous findings (11, 13, 14). Together, these changes in bone formation and endocortical remodeling prevented marrow enlargement and led to significant increases in cortical thickness and cortical BMD in the ABL groups relative to the TPTD group (Table 1). Despite these improvements in cortical bone parameters, no significant changes in biomechanical parameters of bone strength in the femurs of the OVX group were detected, except for the ultimate stress in treated sham animals. This lack of measurable effects may reflect the relatively short duration of treatment. Nevertheless, the intrinsic bone strength parameter data indicate that bone quality was preserved with both treatments.

Thus, as in previous studies, the most striking differences between ABL and TPTD treatments were observed in the cortex. However, no study to our knowledge has attempted to identify differential effects of ABL and TPTD on cortical osteocytes in vivo and in an animal model of estrogen-deficient osteoporosis. In fact, little is known about how long-term intermittent in vivo TPTD or ABL treatment affects osteocytes and their lacunar microenvironment. It is well established that while osteocytes act in a paracrine manner to regulate bone surface remodeling, they also remodel their own lacunar surfaces (PLR), leading to increases or decreases in lacunar area (32, 48–50). Increased lacunar area is generally attributed to osteocytic osteolysis, and such effects are observed in lactating female mice in parallel with an elevation of genes normally associated with bone resorption such as *Mmp13*, *cathepsin K*, and *Acp5* (51, 52). After lactation ceases, lacunar size returns to prelactation levels, with evidence of increased expression of genes associated with bone matrix synthesis and increased bone formation on lacunar surfaces (49).

Here we report that both ABL and TPTD chronic intermittent administration induced significant responses in cortical osteocytes, both in terms of PLR and transcriptomic changes: both reduced significantly the average cortical individual osteocyte lacunar area whereas, despite a pronounced overlap in the changes that occurred in the osteocyte transcriptome, several signaling pathways were differentially affected. These results suggest that although ABL and TPTD activate the same receptor (PTH1R), and initially induce similar signaling responses, the responses of cortical bone, a compartment in which the number of osteocytes relative to bone surfaces is high, differ. Here we show that the transcriptomic response of cortical osteocytes differs and may contribute to the different responses observed here and in previous studies in cortical bone.

While osteocytes tend to respond to ovariectomy with an enlargement of their lacunae (+8.7%), this difference versus sham controls did not reach statistical significance by 2-way ANOVA. However, both TPTD and ABL not only prevented the enlargement associated with ovariectomy but also significantly reduced the LcA in both sham and OVX mice, relative to VEH-treated mice (*P* < 0.001). Thus, osteocytes may exhibit different responses to short-term continuous PTHrP infusion, previously shown to enlarge lacunae (52), and chronic and intermittent ABL and TPTD administration, shown here to significantly reduce lacunar size. This cellular response could reflect a decrease in peri-lacunar resorption, an increase in newly deposited mineralized matrix by the osteocytes, or both. As discussed below, our bulk RNA-Seq results suggest that in fact, similar to their effects on cortical endosteal bone remodeling, chronic and intermittent activation of the PTH1R in osteocytes affect PLR by increasing both osteolysis and matrix deposition, i.e., PLR, and the changes in lacunar area suggest that this increase in PLR occurs with a positive net balance, leading to the reduction of osteocyte LcA.

Analysis of bulk RNA-Seq from highly enriched cortical osteocyte preparations from cortical bone of sham and OVX mice treated with TPTD or ABL showed that the transcriptomic changes induced by treatment were generally similar in sham and OVX mice and between the 2 treatments. Yet, we identified a specific set of genes that responded uniquely to ABL, suggesting differences in downstream signaling and direction or amplitude of change in target genes. Furthermore, among the genes regulated by both ligands, ABL generally exhibited a stronger effect than TPTD. This applied to both osteocyte genes related to the regulation of osteoclastogenesis, such as *Tnfsf11* (*Rankl*) and *Tnfrsf11b* (*Opg*), and genes classically considered “resorption” markers, including the gene encoding *Trap* (*Acp5*), the calcitonin receptor (*Calcr*), or *Dcstamp* for instance. This suggests a shift of some osteocytes to the osteolytic mode, as observed during lactation (62, 63). The same more powerful regulation by ABL was also observed for genes associated with the osteocyte phenotype, such as *Dmp1*, *Phex*, *Sost*, *Pdpn* (*E11*), and *Fgf23*, or the *Pthr1*, *Lrp5*, and *Lrp6* receptors (38). Thus, at the level of the osteocyte, it appears that ABL and TPTD activate both resorption and formation marker genes, reflecting increased PLR activity. Genes within the Wnt and TGF-β signaling pathways were both regulated by TPTD and ABL but here again with significant profile differences between the 2 treatments. Whether these changes occur in different osteocytes, some acquiring the “resorbing” phenotype and others the “matrix forming” phenotype, or whether they reflect a “cyclic” set of changes equivalent to “remodeling” along the lacunar surface (PLR) cannot be determined here, and only single-cell RNA-Seq, a very challenging technique for these deeply embedded cells, may be able to address this important question.

Although ABL and TPTD induced mostly overlapping changes in the genes they regulate, detailed comparisons revealed some relevant signaling pathways uniquely regulated by ABL in both sham and OVX mice, suggesting the possibility of subtle differences between the mechanisms used by ABL and TPTD to alter the osteocyte transcriptome and exert their anabolic activity on cortical bone. Specifically, ABL uniquely upregulated the JAK/STAT and the cytokine-cytokine receptor pathway and uniquely downregulated the IP3 and the chemokine signaling pathways. All 4 of these pathways are directly relevant to GPCR and cytokine receptor signaling, and such changes could therefore elicit specific responses in cortical osteocytes after treatment with ABL, contributing to the differential response of cortical bone observed in this and other studies. Importantly, several of the individual genes identified by the RNA-Seq as being uniquely regulated by ABL are common to several of these 4 pathways and are known to be relevant to bone biology, such as *Lepr*, *Il6*, *Il7*, *Ccr3*, or *Ghr* (53–57). Thus, we conclude that ABL affects the cortical osteocyte transcriptome in a manner subtly different from TPTD, resulting in more beneficial changes in remodeling/modeling and homeostasis of the cortex.

This study has several limitations. Our study involved only female mice, ovariectomized at 12 weeks of age, followed 7 weeks later by a 4-week treatment regimen using a unique dosage of ABL and TPTD (40 μg/kg/d), such that the findings have uncertain generalizability to other models or study designs. Similarly, the bones were collected at a single time point, 18–24 hours after the last TPTD or ABL injection, to isolate RNA and perform bulk RNA-Seq. The selection of these specific time points may have affected several parameters in the skeleton and/or the osteocyte transcriptome. For instance, we observed a lower (although not significant) number of osteoclasts in all OVX groups treated with TPTD or ABL when the opposite was observed in the sham animals and in several studies at earlier time points (26). Some studies have, however, seen unchanged or decreased osteoclast number after chronic TPTD treatment (58, 59). Also, all studies using cortical bone must recognize that the source of cells and the methods used to isolate and enrich for osteocytes have significant limitations. First, it is well established that mouse cortical bone is vascularized, with many micro-channels (60), within which blood vessels, stem cells, and osteoclasts can be found (62). It is therefore difficult to formally attribute transcriptomic changes in RNA extracted from cortical bone solely to osteocytes, even after digestion of the more superficial endosteal and periosteal cell layers. In particular, one cannot strictly attribute the expression of osteoclast marker genes to a change within osteocytes toward an osteoclastic phenotype when using PCR or bulk RNA-Seq. Single-cell RNA-Seq may be able to address this question in the future. Second, the methods used to isolate osteocytes from bone all require the use of sequential digestions with collagenase and EDTA over long periods, possibly affecting the osteocyte transcriptome (61). It is nevertheless reassuring that all osteocyte markers were highly expressed and markedly enriched in our preparations, attesting to the likelihood that RNA was primarily extracted from osteocytes and that the cells did not drastically change their transcriptome during the isolation process.

Despite these limitations, the fact that several pathways are uniquely regulated by ABL in osteocytes, and in particular the JAK/STAT and the IP3 signaling pathways, suggests that ABL and TPTD have subtly distinctive effects on these important bone cells in vivo. Together, bulk RNA-Seq analysis of osteocyte-enriched long bone cell preparations reveals that, although TPTD and ABL have similar anabolic effects, some of the local mechanisms by which they achieve this effect, although mostly shared, are also in part due to activation or repression of distinct signaling cascades, possibly explaining the differences observed in the cortical bone responses to ABL versus TPTD here and in other studies (4, 8, 11, 13).

In conclusion, this study verified that ABL exerts stronger effects than TPTD, even at the same dose, on cortical bone. Our results show that, in vivo, cortical osteocytes respond to ABL and TPTD with an enhanced PLR and a positive balance, similar to the effects of these treatments along the endosteal surfaces and leading to a reduction in the average area of cortical osteocyte lacunae. While cortical osteocytes exhibited significant overlap in the changes in gene expression elicited by ABL and TPTD, responses to the 2 peptides differed in their regulation of several signaling pathways, possibly contributing to the observed differences in cortical responses. Further studies will be necessary to elucidate the role of these pathways and specific genes in the cortical bone responses to ABL and their contribution to the overall skeletal response to treatment.

## Methods

### Animals.

Eight-week-old C57BL6 WT females purchased from Charles River Laboratories were housed under standard environmental conditions (25°C, 50% humidity, 12-hour light/12-hour dark cycle). All mice had free access to filtered water and standard rodent chow diet containing 0.8% calcium and 0.6% phosphate (5058, Pico Lab). Animals were inspected daily for general health and their body weights monitored regularly. Each mouse was assigned a unique number to allow masked analyses. After 2 weeks’ acclimation and at 12 weeks of age, mice underwent bilateral sham or ovariectomy surgery. Seven weeks postsurgery, both groups were injected daily SC for 4 weeks with 0.9% normal saline (VEH), ABL (provided by Radius Health) at 40 μg/kg/d, or TPTD (Bachem) at 40 μg/kg/d, providing a direct potency comparison. Mice were sacrificed at 23 weeks of age, 24 hours after the final peptide injection (Supplemental Figure 1A). Uterine horns were weighed for atrophy to confirm successful ovariectomy.

The dosage of 40 μg/kg/d was selected based on the following: the human dose is 20 μg/kg/d for TPTD and 80 μg/kg/d for ABL. Since in this study we wanted to compare the 2 agents at the same dose, we selected a common dose that would be active but neither overdosing TPTD nor underdosing ABL. In mice TPTD has been used and shown to be active on bone within a range between 10 and 100 μg/kg/d; for ABL most animal studies have been done at dosages between 10 and 80 μg/kg/d (62). Based on this information, the dose of 40 μg/kg/d was selected.

### μCT analysis.

A high-resolution desktop micro-tomographic imaging system (μCT40, Scanco Medical AG) was used to assess trabecular bone architecture and mineral density in the L5 vertebral body and cortical bone architecture in the diaphysis of the tibia and femur. Scans were acquired using a 10 μm^3^ isotropic voxel size, 70 kVP, 114 mAs, and 200 ms integration time. μCT scanning and analysis were performed according to recommended guidelines (63). For trabecular analysis, a region of interest was semimanually contoured in the endocortical region of the L5 vertebral body (100 μm inferior to the cranial endplate to 100 μm superior to the caudal endplate) Trabecular bone within this region was segmented from soft tissue using a threshold of 400 mg HA/cm^3^. The Scanco trabecular morphology evaluation script was used to measure trabecular bone volume fraction (Tb.BV/TV, %), trabecular bone mineral density (Tb.BMD, mg HA/cm^3^), trabecular number (Tb.N, per mm), trabecular thickness (Tb.Th, μm), trabecular separation (Tb.Sp, μm), structural model index, and connectivity density (Conn.D, per mm). Cortical bone was assessed in 500 μm–long regions (50 transverse slices) at the femoral mid-diaphysis and tibial diaphysis (2 mm superior to the distal tibiofibular junction). Images within the cortical region of interest were segmented using a threshold of 700 mg HA/cm^3^, and then the Scanco midshaft evaluation script was used to measure total cross-sectional area (bone + medullary area) (Tt.Ar, mm^2^), cortical bone area (Ct.Ar, mm^2^), medullary area (Ma.Ar, mm^2^), bone area fraction (Ct.Ar/Tt.Ar, %), cortical tissue mineral density (Ct.TMD, mg HA/cm^3^), cortical thickness (Ct.Th, mm), as well as maximum, minimum, and polar moments of inertia (Imax, Imin, and pMOI, mm^4^).

### Bone histomorphometry analysis.

Mice were injected with 20 mg/kg calcein and demeclocycline (MilliporeSigma) on days 8 and 2 before sacrifice, respectively, to fluorescently label newly formed bone. Vertebrae (L3–L5) and tibiae were fixed in 70% ethanol and embedded in methylmethacrylate (MMA). Vertebral histomorphometric data were obtained under 200× original magnification in a 1.3 × 1.8 mm region away from the growth plate to avoid including primary spongiosa. The tibial diaphysis was bisected with an IsoMet 1000 Precision Saw (Buehler) at the midpoint between the proximal and distal tibiofibular junctions. Tibia cortical bone histomorphometric data were obtained under 100× original magnification from cross sections obtained in the exact same region of interest as the μCT and the BSEM measurements (see *BSEM* below). Consecutive cross sections of the distal tibia and frontal sections of the vertebral body (4 μm thickness) were stained with von Kossa and toluidine blue for the analysis of cellular parameters and osteoid. Bone sections were viewed with a Nikon E800 microscope equipped with Olympus DP71 digital camera. Images were captured using Olympus CellSens software.

Structural parameters for vertebral body included bone volume per tissue volume (BV/TV), trabecular thickness (Tb.Th), trabecular number (Tb.N), and trabecular separation (Tb.Sp). Structural parameters for the tibial diaphysis included total area (Ct.T.Ar), cortical bone area (Ct.B.Ar), cortical marrow area (Ma.Ar), cortical BV/TV (Ct.BV/TV), cortical thickness (Ct.Th), and endocortical perimeter (Ec.Pm). Periosteal perimeter (Ps.Pm) was measured from a single toluidine blue section while all the other cortical and trabecular structural parameters were determined by averaging the results of toluidine blue and von Kossa staining. Cellular parameters measured in vertebral trabecular bone included osteoblast surface per bone surface (Ob.S/BS), osteoblast number per bone perimeter (Ob.N/B.Pm), osteoclast surface per bone surface (Oc.S/BS), osteoclast number per bone perimeter (Oc.N/B.Pm), osteoid surface per bone surface (OS/BS), and osteoid thickness (O.Th). Additional unstained sections were used for dynamic parameter measurements, including MAR, MS/BS, BFR/BS, and BFR/BV. OsteoMeasure analyzing software (Osteometrics Inc.) was used to generate and calculate the data. All the parameters were obtained under a blinded protocol and presented according to standardized nomenclature (64).

### Femur bending strength and biomechanics.

Femurs were mechanically tested in 3-point bending using an electrical force material testing machine (Electroforce 3230, Bose Corporation). The bending fixture had a bottom span length of 8 mm. The test was performed in displacement control, with the applied load moving at a rate of 0.03 mm/s. Force and displacement data were collected at 50 Hz. Bones were positioned in the same orientation during testing with the cranial surface resting on the supports and loaded in tension. Bending rigidity (EI, N-mm^2^), apparent bending modulus (E_app_, MPa), ultimate moment (M_ult_, N-mm), apparent ultimate stress (σ_app_, MPa), work to fracture (W_frac_, mJ), and apparent toughness (U_app_, mJ/mm^3^) were calculated on the basis on the force and displacement data from the tests and the midshaft geometry measured with μCT. W_frac_, reflecting the energy required to fracture the femur, was calculated as the area under the force-displacement curve using the Riemann sum method. EI was calculated using the linear portion of the force-displacement curve. The minimum moment of inertia was used when calculating the apparent bending modulus and bending strength (ultimate stress).

### Biochemical markers of bone turnover.

Fasting serum levels of the bone formation marker P1NP and the bone resorption marker CTX were measured using commercial kits from Immunodiagnostic Systems.

### BSEM.

BSEM imaging was used to quantify osteocyte lacunae in tibia midshaft cortical bone. Measurements of lacunar density and area were performed on images acquired by a scanning electron microscope equipped with a backscattered electron detector operated with an accelerating voltage of 20 kV, a working distance of 10.0 mm, aperture size of 30.00 μm, and 500× original magnification (SEM, Supra 55 VP, Zeiss, Center for Nanoscale Systems in Harvard University). MMA-embedded tibia sample blocks were sequentially polished with silicon carbide sandpaper of increasing grit number (240, 600, 2,400, 6,000, 8,000 grit) and alumina polishing micromesh cloth of 12,000 grit (Scientific Instrument Services Inc.). Blocks were sputter-coated with 5 nm of carbon-supported platinum and palladium alloy (Leica Microsystems Inc.) and mounted and fixed with aluminum conductive tape (Ted Pella, Inc.). BSEM images were taken at a standardized tibial midshaft area located 4.5 mm distal from the proximal tibia-fibula junction, the same standardized region used for μCT and histomorphometry analyses. Images were thresholded using default method in ImageJ 1.52d (NIH) and analyzed using Fiji software, to measure mean LcA (mm^2^) and LcD (#/mm^2^), this latter reflecting the number of lacunae per area of mineralized bone analyzed.

### RNA isolation from osteocyte-enriched fraction of long bone.

Osteocyte RNA was isolated from mouse long bones using a modified method described in Stern et al. (65). Briefly, tibiae and femurs were aseptically dissected, with epiphyses cut off and diaphyses flushed with PBS and centrifuged to remove bone marrow. Cortical midshafts were washed in α-MEM (Gibco Laboratories) containing 1% penicillin and streptomycin (Gibco Laboratories) and cut into pieces (1–2 mm) that were subjected to 3 sequential digestions to remove periosteum, fibroblasts, osteoblasts, osteoclasts, and other adherent cells. For the first digestion, bone pieces were incubated for 25 minutes at 37°C with a collagenase solution (0.25 mg/mL collagenase type I and 0.75 mg/mL collagenase type II in α-MEM supplemented with 0.1% BSA and 25 mM HEPES) (Worthington Biochemical Corporation) on an orbital shaker (Innova 4000 incubator shaker, Marshall Scientific) set to 100 rpm. Following a PBS wash, 2 sequential collagenase digestions were performed before incubating the bone pieces with 5 mM EDTA/1% BSA. The EDTA solution was removed and discarded, the bone pieces were incubated twice with collagenase/trypsin Solution (2 mg/mL collagenase type II and 1% trypsin in α-MEM supplemented with 25 mM HEPES) for 25 minutes at 37°C. These digestions were collected and defined as osteoblast-enriched population. The digested long bone particles (osteocyte-enriched population) were homogenized using a tissue homogenizer (Tekmar Tissumizer Homogenizer SDT-1810) in TRIzol reagent (Thermo Fisher Scientific), and total RNA was isolated using RNeasy Plus Micro Kit (QIAGEN) following the manufacturer’s instructions.

### RNA-Seq.

Experimental duplicates were performed for each condition. Sample libraries were prepared using the Ion Ampliseq Transcriptome Mouse Gene Expression Panel (Thermo Fisher Scientific) according to the manufacturer’s protocol (66). Briefly, 10 ng of total DNA was reverse-transcribed before PCR amplification of the resulting cDNA. The amplicons were partially digested before ligation of adapters and multiplex barcodes. Ligation products were purified with AMPure XP beads (Beckman Coulter) and quantified using the Ion Library TaqMan Quantitation Kit (Thermo Fisher Scientific). Libraries were normalized to 70 pM concentration and combined in batches of no more than 8 samples for further processing. Templating and Ion 540 chip loading were performed on the Ion Chef (Thermo Fisher Scientific) followed by sequencing on the Ion S5 (Thermo Fisher Scientific) according to the manufacturer’s instructions. Raw reads were processed using the Torrent Suite analysis pipeline to the mm10 mouse genome assembly using TMAP. Raw counts assigned to genes were generated using the RNA-Seq Analysis plugin in Torrent Suite and normalized by R package DESeq2 (v4.0) or EdgeR (v3.32.1) by R software (v3.6.2) (67, 68). DEGs were identified by the criterion of FDR < 0.05. R package ClusterProfiler (v3.14.3) was employed to analyze the GO and KEGG to annotate the DEGs, the results of which were visualized by bubble plot (69). GSEA was employed to identify related pathways (70).

### Real-time quantitative reverse transcription PCR.

Total RNA from osteoblast- and osteocyte-enriched fractions was used to synthesize first-strand cDNA with SuperScript II (Invitrogen, Thermo Fisher Scientific) for reverse transcription PCR. mRNA levels encoding each gene of interest were normalized to actin mRNA in the same sample, and the relative expression of the genes of interest was determined using the formula of Livak and Schmittgen (71).

### Western analysis.

After 14 days of culture in α-MEM, 10% FBS, and 1% Pen/Strep (all Life Technologies) at 37°C, Ocy454 cells were treated with TPTD 5 nM, ABL 5 nM, or vehicle for 5 minutes to 3 hours. The Ocy454 cells were obtained from Paola Divieti Pajevic from the MGH Center for Skeletal Research, Boston, Massachusetts, USA. Total proteins (10 μg) were resolved by SDS-PAGE under reducing conditions. Immunodetection was performed with S246 phospho-HDAC4/ S259 phospho-HDAC5 (Cell Signaling Technology 3443), phospho-CRCT2 (Abcam, ab203187), HDAC4 (Abcam, ab12172), HDAC5 (Abcam, ab55403), or GAPDH (Santa Cruz Biotechnology, SC-32233). GAPDH was used as loading control. Immunoreactive proteins were detected using SuperSignal West Dura Extended Duration Substrate (Thermo Fisher Scientific, 34075). Films were scanned (Canon, CanoScan 9000F) and saved as 600 dpi color images.

### Statistics.

All data are shown as mean ± SEM unless otherwise specified. Two-way ANOVA was performed to test the impact of different drug treatments and ovariectomy, followed by Fisher’s LSD test to determine the significance between all groups. GraphPad Prism 9 software was used for statistical analyses. *P* < 0.05 was considered statistically significant.

### Study approval.

All surgical procedures and treatments were approved by Harvard Medical School Institutional Animal Care and Use Committee.

### Data availability.

Data are available in the Supporting Data Values XLS file and from the corresponding author upon request. Bulk RNA-Seq data have been deposited in the NCBI BioProject (PRJNA1009097).

## Author contributions

RB, BL, and FG designed the study. ZL, SL, CZ, and JZ performed experiments. ZL, SL, DJB, and DH performed measurements. RB, FG, and ZL interpreted the data. JZ performed the bioinformatic analysis. JZ, FG, and RB analyzed the RNA-Seq data. MLB and DJB interpreted the biomechanics data. ZL, JZ, FG, and RB wrote the manuscript. PK, BL, MLB, FG, and RB edited the manuscript.

## Supplementary Material

Supplemental data

Supporting data values

## Figures and Tables

**Table 1:**
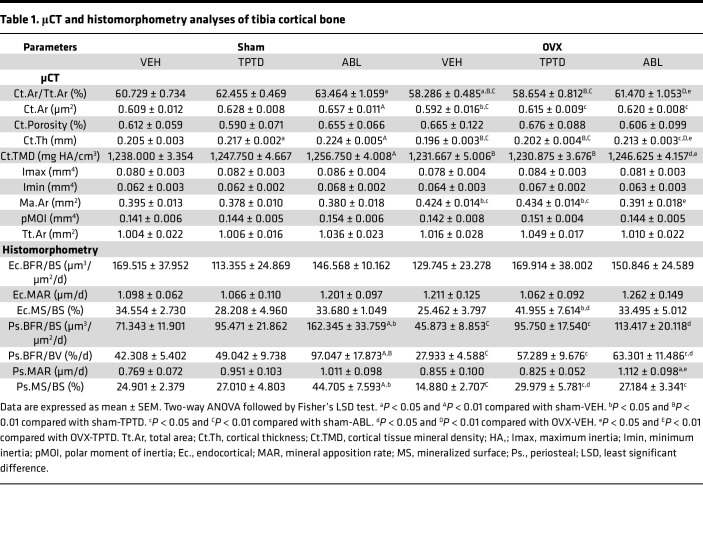
μCT and histomorphometry analyses of tibia cortical bone

## Version 1 10/23/2023

Electronic publication
