# Supplementary material for: Comparative study in estrogen-depleted mice identifies skeletal and osteocyte transcriptomic responses to abaloparatide and teriparatide
Source: JCI Insight. 2023 Oct 23;8(20):e161932. doi: 10.1172/jci.insight.161932 (PMC10619488; doi:10.1172/jci.insight.161932)
Supplement: Supplemental data [file jciinsight-8-161932-s074.pdf]

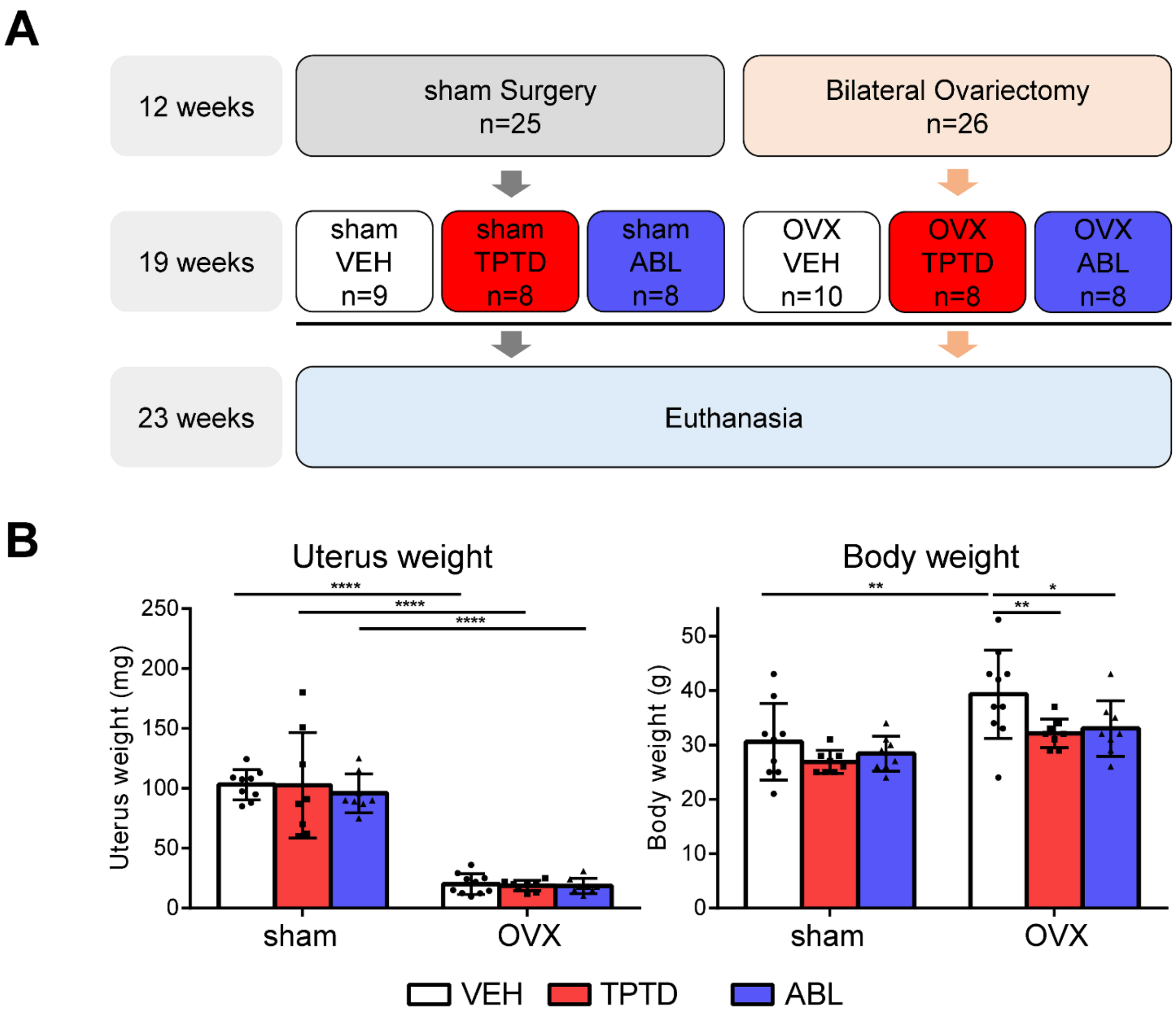

Figure S1. *Flow chart and quality control of the study*. A. Study design. B. Uterus and body weight in sham and OVX groups. Two-way ANOVA followed by Fisher's LSD test. *p<0.05, **p<0.01, ***p<0.001, ****p<0.0001.

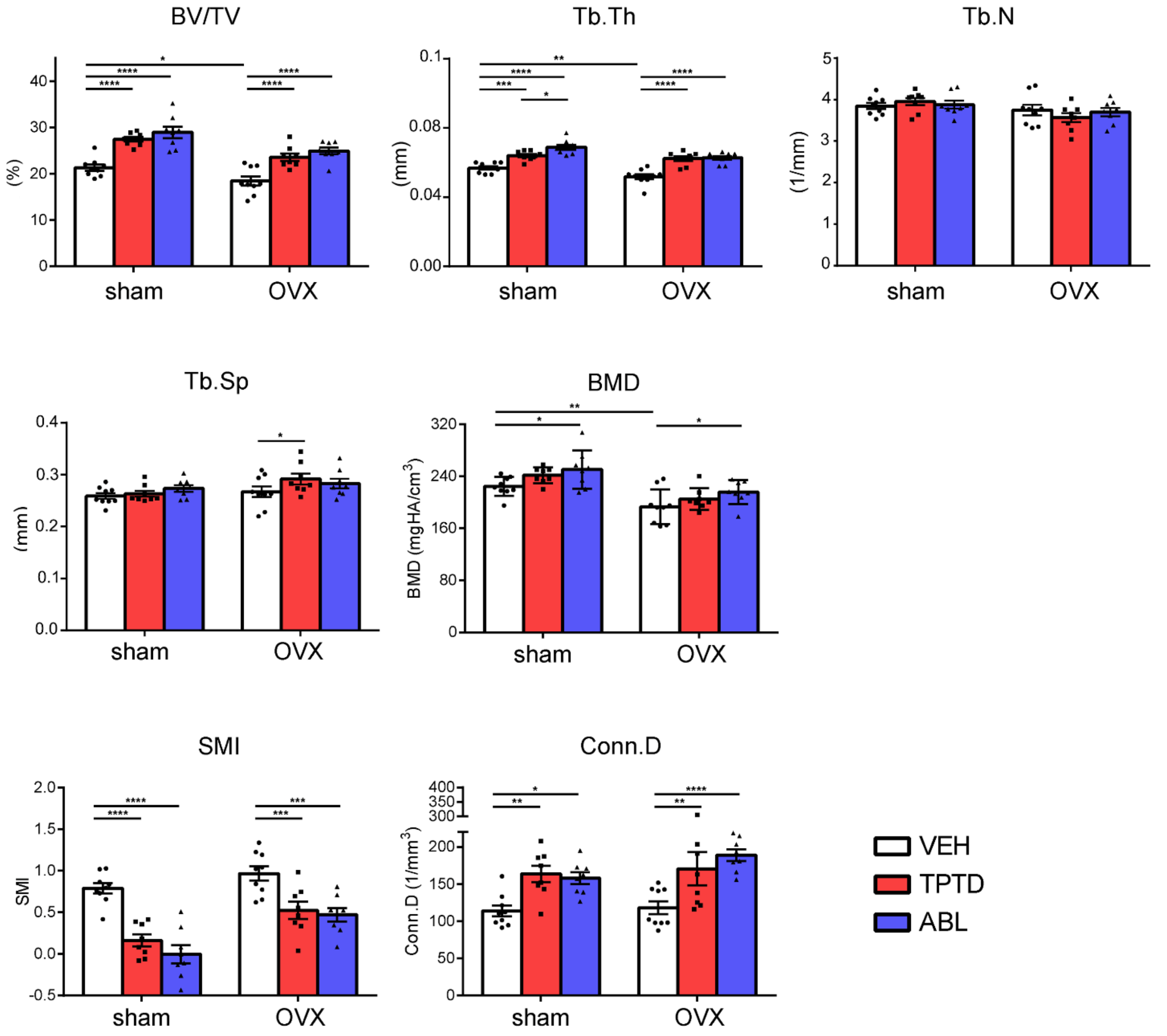

Figure S2. *Trabecular bone responses.* μCT analysis of trabecular bone at L5 vertebrae in sham and OVX groups treated with vehicle, TPTD, or ABL. Data are expressed as mean ± SEM. Individual dots represents the number of mice in each group. Two-way ANOVA followed by Fisher's LSD test. *p<0.05, **p<0.01, ***p<0.001, ****p<0.0001.

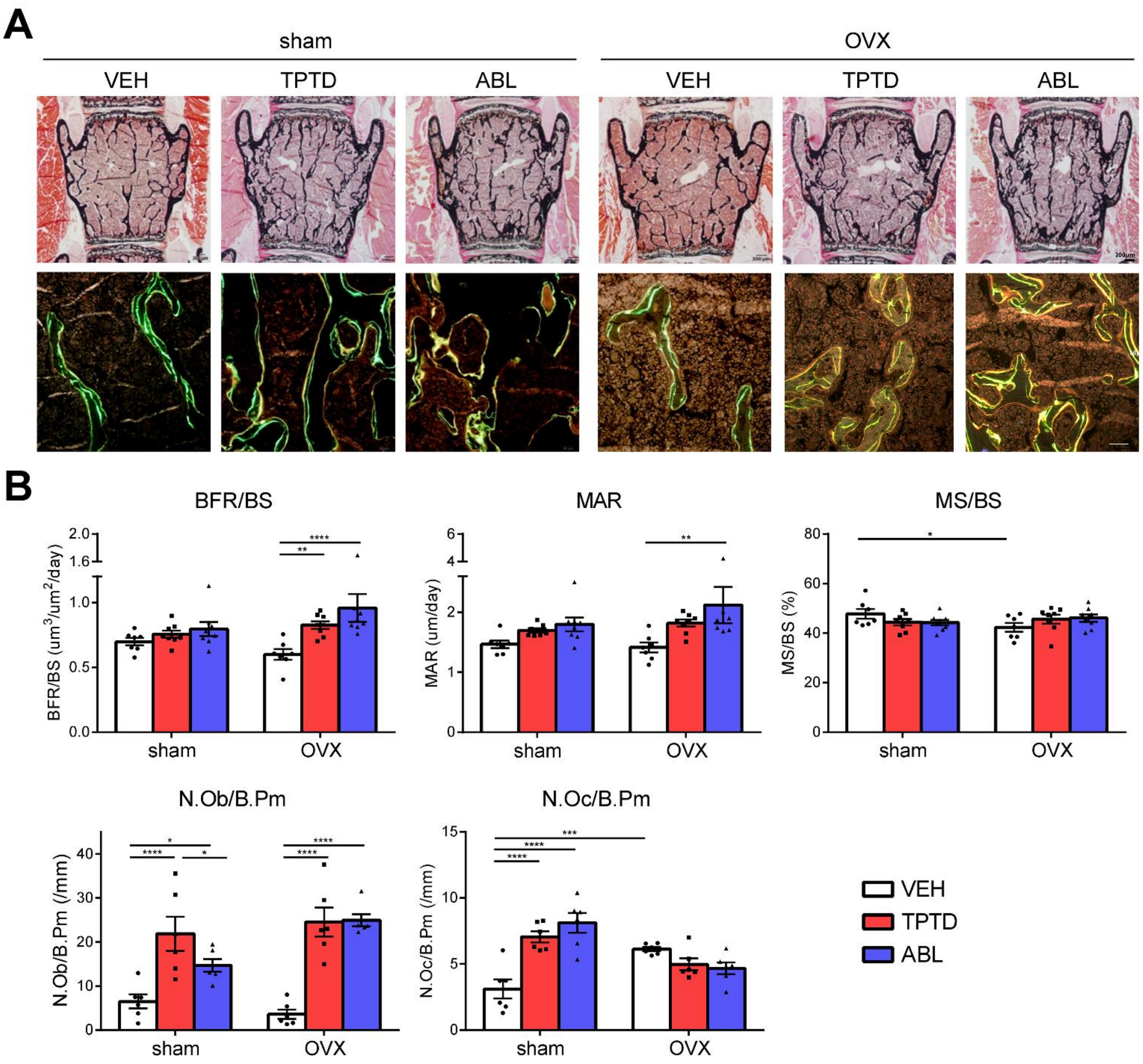

Figure S3. *Trabecular bone responses.* Histomorphometry analysis of trabecular bone at L5 vertebrae in sham and OVX groups treated with vehicle, TPTD or ABL. **A)** Representative images of Von Kossa Staining (upper panel) and calcein double labeling (lower panel). **B)** Dynamic and cellular histomorphometry analysis. Data are expressed as mean ± SEM. Individual dots represents the number of mice in each group. Two-way ANOVA followed by Fisher's LSD test. *p<0.05, **p<0.01, ***p<0.001, ****p<0.0001.

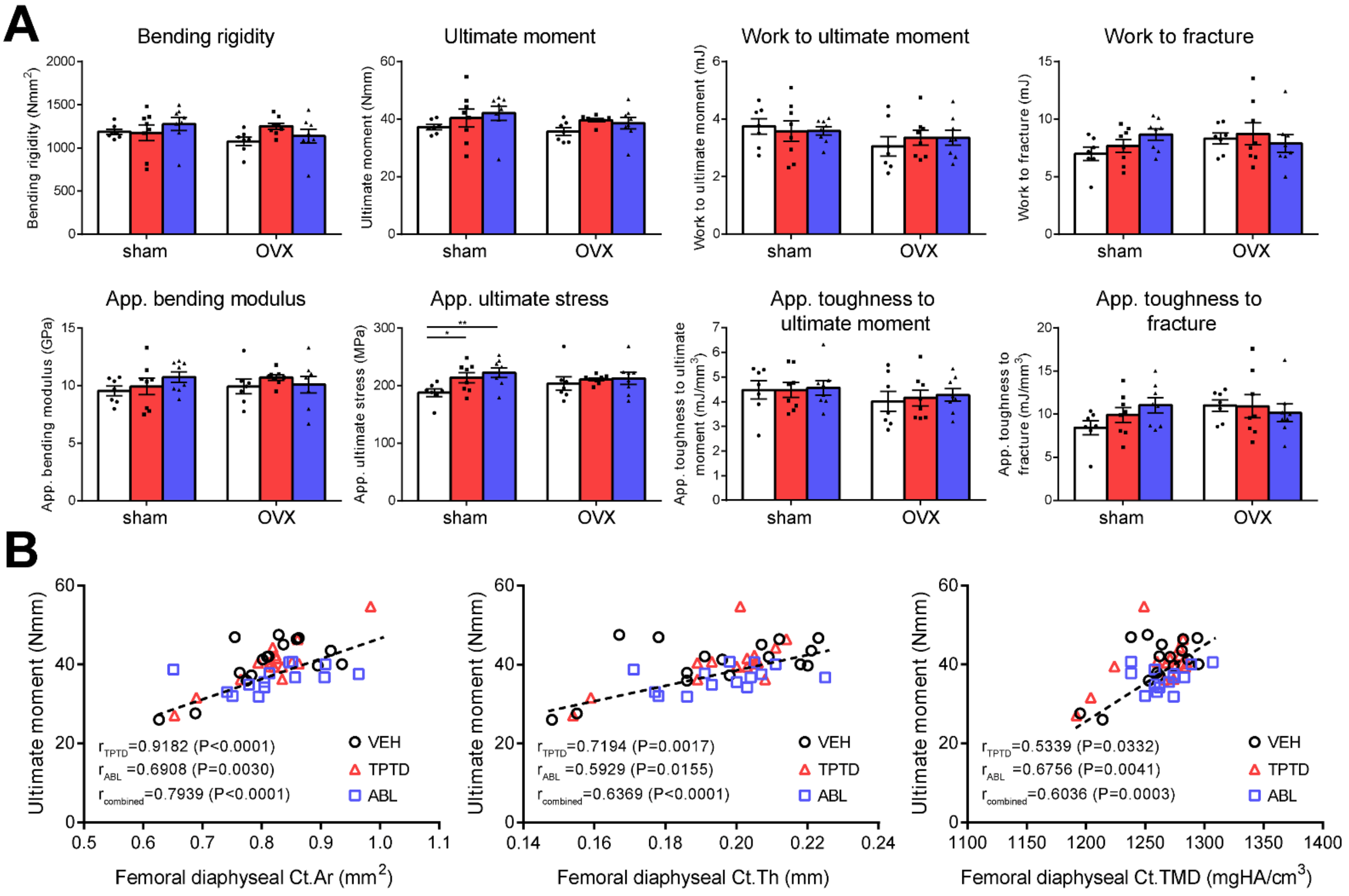

Figure S4. *Biomechanics analysis of femoral mid-diaphysis*. **A)** Biomechanical parameters in sham and OVX groups treated with vehicle, TPTD or ABL. Data are expressed as mean ± SEM. Individual dots represents the number of mice in each group. Two-way ANOVA followed by Fisher's LSD test. *p<0.05, **p<0.01. **B)** Correlations between µCT-derived femoral structural parameters and ultimate moment evaluated by linear regression analysis.

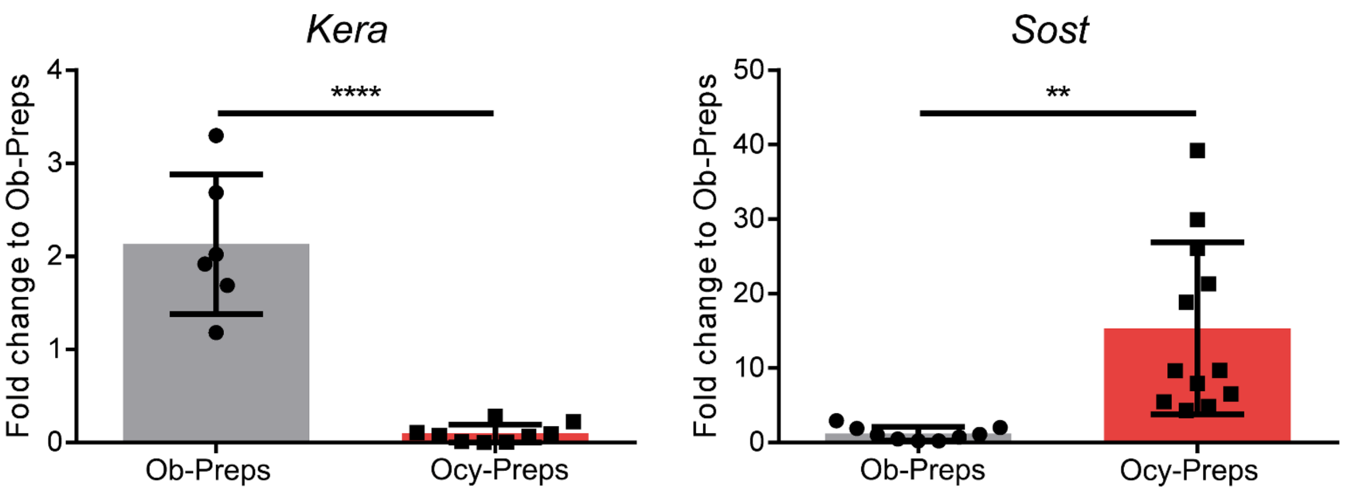

Figure S5. *Validation of osteocyte-enriched population.* qRT-PCR analysis of *Kera* and *Sost* expression in OB- and Ocy-enriched fractions. Data are expressed as mean ± SD. Individual dots represents the number of mice in each group. Unpaired Student t-test; **p<0.01, ****p<0.0001.

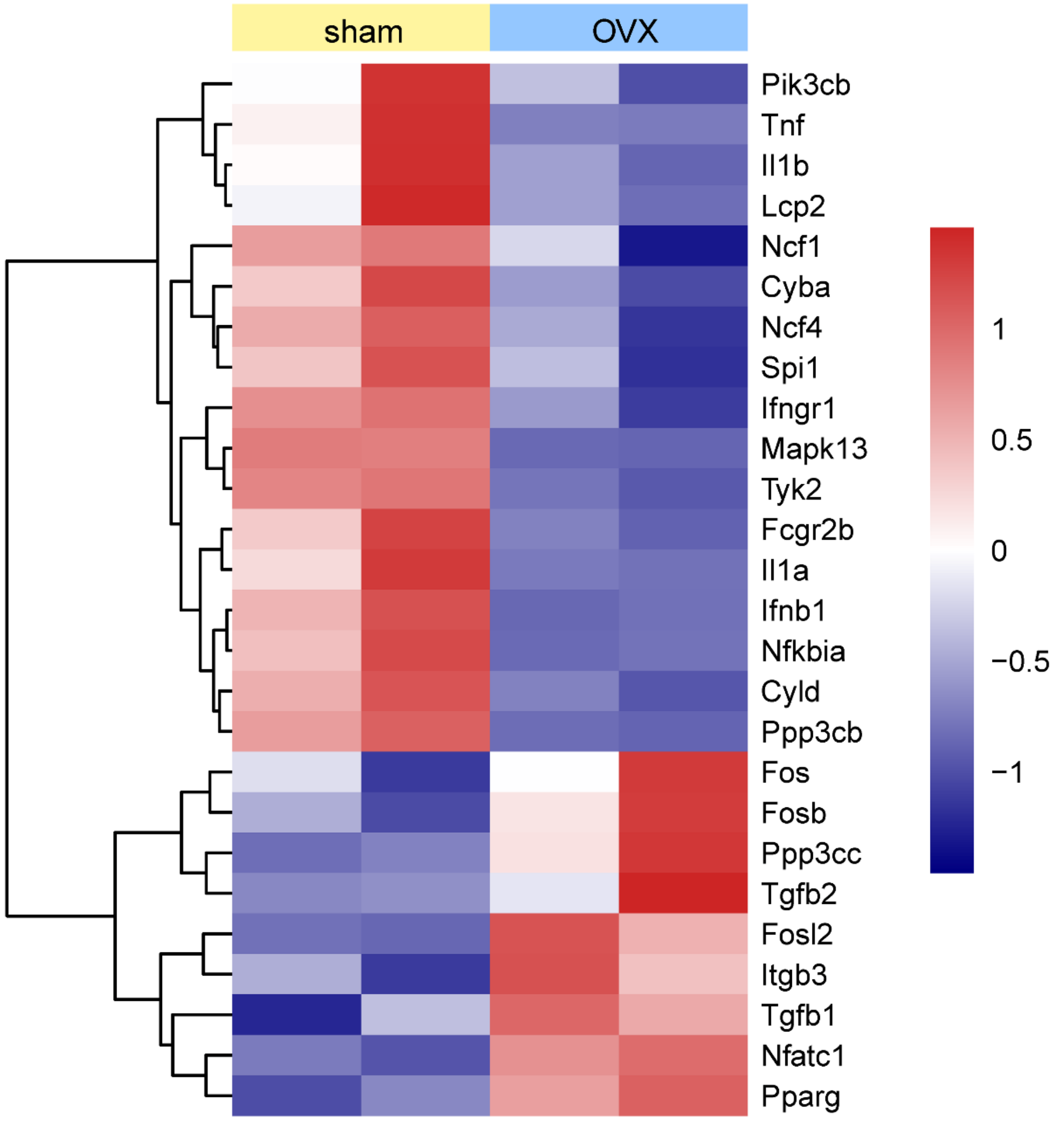

Figure S6. *Bulk RNA-seq*. HeatMap of genes relevant to osteoclast differentiation.

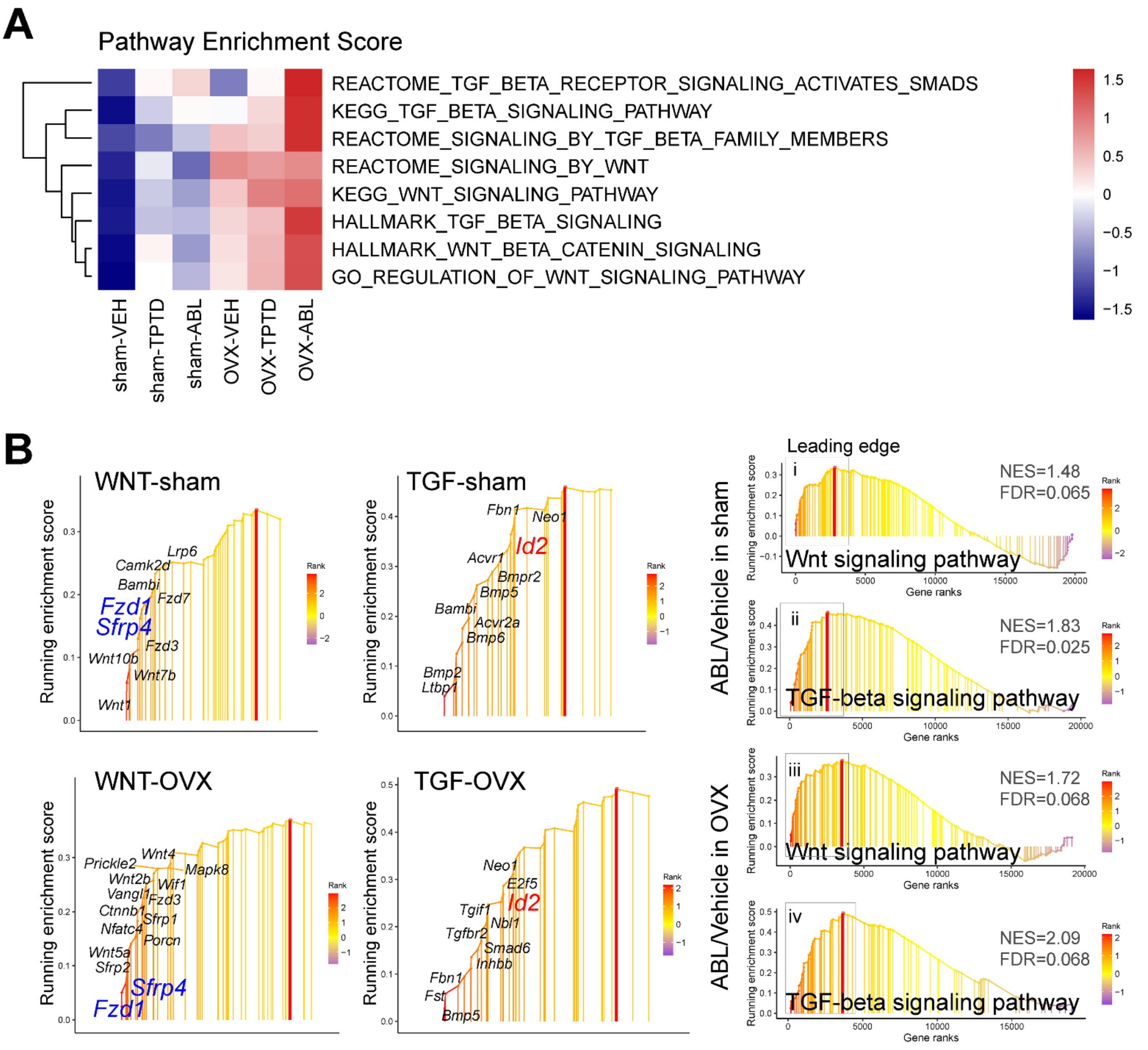

Figure S7. *Bulk RNA-seq.* Wnt and TGF signaling regulation by TPT or ABL4 treatment in sham or OVX groups. **A)** Pathway enrichment score estimated by ssGSEA. **B)** *Sfrp4* and *Fzd1* (blue) are genes belonging to the Wnt signaling regulated by both TPTD and ABL. *Id2* (red), a gene of the TGF signaling pathway is regulated only by ABL.

Table S1. μCT and histomorphometry analyses of L5 vertebrae.

Parameters	sham			OVX		
	VEH	TPTD	ABL	VEH	TPTD	ABL
**m-CT**						
BV/TV (%)	21.314±0.668	27.459±0.487^{A}	28.965±1.247^{A}	18.427±0.972^{aBC}	23.583±0.813^{BCD}	24.936±0.742^{AbCD}
BMD (mgHA/cm^3)	224.333±4.910	241.375±4.338	250.500±10.428^{a}	193.111±8.832^{ABC}	205.000±5.880^{BC}	215.625±6.478^{bCd}
BS/BV (mm^2/mm^3)	40.481±0.762	35.136±0.661^{A}	32.806±0.661^{A}	44.857±1.564^{ABC}	37.053±1.033^{aCD}	36.799±0.800^{acD}
Conn.D. ($1/mm^3$)	113.678±7.323	163.550±11.063^{A}	157.925±7.813^{a}	117.978±8.597^{Bc}	170.525±22.378^{AD}	188.838±7.046^{AD}
SMI	0.790±0.062	0.164±0.072^{A}	-0.004±0.108^{A}	0.968±0.086^{BC}	0.525±0.102^{aBCD}	0.472±0.082^{abCD}
Tb.N (1/mm)	3.847±0.074	3.953±0.089	3.879±0.089	3.748±0.124	3.564±0.109^{bc}	3.700±0.100
Tb.Th (mm)	0.057±0.001	0.064±0.001^{A}	0.069±0.001^{Ab}	0.052±0.001^{ABC}	0.062±0.001^{ACD}	0.063±0.001^{ACD}
Tb.Sp (mm)	0.259±0.006	0.263±0.006	0.273±0.06	0.267±0.010	0.292±0.010^{Acd}	0.283±0.009^{ab}
**Histomorphometry**						
BFR/BS ($\mu m^3/\mu m^2$/day)	0.679±0.029	0.755±0.028	0.795±0.053^{d}	0.598±0.041^{c}	0.825±0.030^{D}	0.958±0.107^{AbcD}
BFR/BV (%/day)	3.667±0.161	4.699±0.255^{a}	4.301±0.320^{d}	3.228±0.242^{Bc}	4.804±0.203^{aD}	5.432±0.672^{AcD}
MAR (μm/day)	1.466±0.061	1.699±0.034	1.799±0.114	1.414±0.082	1.820±0.059	2.121±0.303^{Abd}
MS/BS (%)	47.780±1.951	44.426±1.260	44.233±1.159	42.320±1.770^{a}	45.604±1.809	46.068±1.493
N.Ob/B.Pm (/mm)	6.474±1.597	21.875±3.878^{A}	14.703±1.442^{ab}	3.591±1.069^{BC}	24.550±3.258^{ACD}	24.951±1.377^{ACD}
N.Oc/B.Pm (/mm)	3.107±0.717	7.057±0.427^{A}	8.101±0.750^{A}	6.123±0.163^{Ac}	4.965±0.451^{abC}	4.671±0.450^{abC}
Ob.S/BS (%)	8.792±2.022	29.478±5.158^{A}	20.364±1.877^{ab}	4.884±1.402^{BC}	30.794±3.981^{AcD}	33.359±1.368^{ACD}
Oc.S/BS (%)	5.824±1.338	14.951±1.116^{A}	17.636±2.044^{a}	12.411±0.599^{BC}	9.759±1.032^{Bc}	9.504±1.021^{Bc}
OS/BS (%)	4.939±1.655	14.082±4.139^{a}	8.972±1.429^{G}	4.344±0.657^{B}	14.379±2.838^{AD}	18.542±2.146^{ACD}
O.Th (μm)	3.130±0.142	3.090±0.151	3.035±0.112	3.046±0.165	2.948±0.069	3.136±0.151

Data are expressed as Mean±SEM. Two-Way ANOVA followed by Fisher's LSD test. a=P<0.05 and A=P<0.01 compared to sham-VEH; b=P<0.05 and B=P<0.01 compared to sham-TPTD; c=P<0.05 and C=P<0.01 compared to sham-ABL; d=P<0.05 and D=P<0.01 compared to OVX-VEH; e=P<0.05 and E=P<0.01 compared to OVX-TPTD.

Table S2. Serum bone formation and resorption markers.

Parameters	sham			OVX		
	VEH	TPTD	ABL	VEH	TPTD	ABL
CTX	19.221±3.089	20.135±0.744	16.054±0.980	26.289±4.729[c]	22.389±2.519	19.463±1.462
P1NP	37.323±3.912	33.283±2.672	22.569±2.022[a]	52.966±8.200[aBC]	51.955±4.037[abC]	45.279±4.294[bC]

Data are expressed as Mean±SEM. Two-WAY ANOVA followed by Fisher's LSD test. a=P<0.05 and A=P<0.01 compared to sham-VEH; b=P<0.05 and B=P<0.01 compared to sham-TPTD; c=P<0.05 and C=P<0.01 compared to sham-ABL; d=P<0.05 and D=P<0.01 compared to OVX-VEH; e=P<0.05 and E=P<0.01 compared to OVX-TPTD.

Table S3. Femur Biomechanical analysis.

Parameters	sham			OVX		
	VEH	TPTD	ABL	VEH	TPTD	ABL
App. bending modulus (GPa)	9.551±0.416	9.939±0.712	10.749±0.466	9.937±0.630	10.713±0.228	10.109±0.725
App. ultimate stress (MPa)	188.096±6.605	214.030±8.712[a]	222.671±8.473[A]	203.947±11.740	211.193±2.698	212.706±10.378
App. toughness to fracture (mJ/mm^3)	8.417±0.808	9.901±0.880	11.041±0.873	11.006±0.659	10.921±1.336	10.160±1.016
App. toughness to ultimate moment (mJ/mm3)	4.479±0.369	4.473±0.299	4.561±0.297	4.019±0.408	4.155±0.318	4.275±0.258
Bending rigidity (Nmm^2)	1186.857±30.467	1176.375±91.965	1276.750±76.553	1076.429±49.277[c]	1252.500±36.819	1139.375±79.433
Ultimate moment (Nmm)	37.257±0.912	40.400±3.078[e]	42.100±2.481	35.671±1.326[c]	39.675±0.564	38.638±1.987
Work to fracture (mJ)	6.994±0.576	7.693±0.563	8.674±0.492	8.317±0.479	8.720±0.955	7.891±0.769
Work to ultimate moment (mJ)	3.746±0.270	3.579±0.351	3.590±0.144	3.053±0.338	3.353±0.257	3.351±0.258

Data are expressed as Mean±SEM. Two-WAY ANOVA followed by Fisher's LSD test. a=P<0.05 and A=P<0.01 compared to sham-VEH; b=P<0.05 and B=P<0.01 compared to sham-TPTD; c=P<0.05 and C=P<0.01 compared to sham-ABL; d=P<0.05 and D=P<0.01 compared to OVX-VEH; e=P<0.05 and E=P<0.01 compared to OVX-TPTD.

T**able S4.** BSEM analysis of the osteocyte lacunar size.

**Parameters**	**sham**			**OVX**		
	**VEH**	**TPTD**	**ABL**	**VEH**	**TPTD**	**ABL**
%LcA	1.303±0.068	1.262±0.102	1.184±0.095	1.369±0.091	1.051±0.079[ad]	1.17±0.053
LcD (10^4/μm^2)	4.394±0.286	4.766±0.335	4.926±0.372	4.234±0.235	3.848±0.33[c]	4.487±0.136
LcA (μm^2)	29.855±1.060	26.583±1.266[A]	24.029±0.717	32.449±1.861[BC]	27.544±0.801[cD]	26.111±1.01[aD]
Freq of LcA <30 μm^2	53.575±3.637	65.641±3.552[a]	71.119±2.596[A]	50.292±4.699[BC]	65.263±2.576[aD]	67.39±3.232[AD]
Freq of LcA ≥30 μm^2	46.59±3.668	34.359±3.552[a]	28.356±2.51[A]	49.483±4.728[BC]	34.737±2.576[aD]	32.61±3.232[AD]
Freq of LcA <20 μm^2	25.579±2.567	32.655±4.963	39.654±2.191[A]	18.193±1.9[BC]	28.776±3.704[cd]	35.835±1.356[aD]
Freq of LcA 20-30 μm^2	27.996±1.858	32.986±2.251	31.466±1.772	32.099±4.299	36.32±2.792[a]	31.555±2.239
Freq of LcA 30-40 μm^2	26.411±2.411	21.146±2.101	20.973±1.7	26.158±2.836	23.283±1.779	19.689±2.337[ad]
Freq of LcA 40-50 μm^2	13.023±1.992	8.452±1.345	5.757±0.684[a]	11.511±3.218[c]	7.968±1.815[e]	8.178±1.507
Freq of LcA 50-60 μm^2	5.476±0.879	2.578±0.758[a]	0.591±0.31[A]	6.55±1.807[BC]	2.114±0.506[aD]	2.47±0.935[aD]
Freq of LcA ≥60 μm^2	1.89±0.645	2.183±0.682	1.035±0.546	5.34±2.344[ac]	1.835±0.48[d]	2.219±0.96

Data are expressed as Mean±SEM. Two-Way ANOVA followed by Fisher's LSD test. a=P<0.05 and A=P<0.01 compared with sham-VEH; b=P<0.05 and B=P<0.01 compared with sham-TPTD; c=P<0.05 and C=P<0.01 compared with sham-ABL; d=P<0.05 and D=P<0.01 compared with OVX-VEH; e=P<0.05 and E=P<0.01 compared with OVX-TPTD.
